# Transcriptional Signatures of Tau and Amyloid Neuropathology

**DOI:** 10.1016/j.celrep.2020.01.063

**Published:** 2020-02-11

**Authors:** Isabel Castanho, Tracey K. Murray, Eilis Hannon, Aaron Jeffries, Emma Walker, Emma Laing, Hedley Baulf, Joshua Harvey, Lauren Bradshaw, Andrew Randall, Karen Moore, Paul O’Neill, Katie Lunnon, David A. Collier, Zeshan Ahmed, Michael J. O’Neill, Jonathan Mill

**Affiliations:** 1 Institute of Biomedical and Clinical Sciences, University of Exeter Medical School, University of Exeter, Exeter EX2 5DW, UK; 2 Eli Lilly & Co., Erl Wood Manor, Sunninghill Road, Windlesham GU20 6PH, UK

**Keywords:** tau, amyloid, transgenic model, Alzheimer’s disease, gene expression, neuropathology, entorhinal cortex, hippocampus, RNA-seq

## Abstract

Alzheimer’s disease (AD) is associated with the intracellular aggregation of hyperphosphorylated tau and the accumulation of β-amyloid in the neocortex. We use transgenic mice harboring human tau (rTg4510) and amyloid precursor protein (J20) mutations to investigate transcriptional changes associated with the progression of tau and amyloid pathology. rTg4510 mice are characterized by widespread transcriptional differences in the entorhinal cortex with changes paralleling neuropathological burden across multiple brain regions. Differentially expressed transcripts overlap with genes identified in genetic studies of familial and sporadic AD. Systems-level analyses identify discrete co-expression networks associated with the progressive accumulation of tau that are enriched for genes and pathways previously implicated in AD pathology and overlap with co-expression networks identified in human AD cortex. Our data provide further evidence for an immune-response component in the accumulation of tau and reveal molecular pathways associated with the progression of AD neuropathology.

## Highlights

• Mice with human tau and amyloid mutations develop progressive neuropathology
• We identify transcriptional changes associated with tau and amyloid pathology
• Discrete co-expression networks are associated with the progression of tau pathology
• These are enriched for genes and pathways implicated in the onset of human AD

## Graphical Abstract

**Figure:**
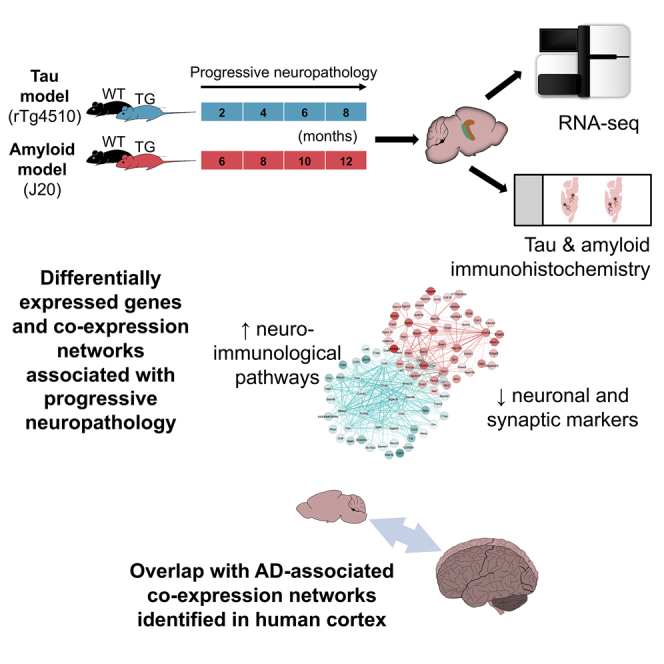

## Teaser

Castanho et al. use transgenic mice harboring human tau and amyloid precursor protein mutations to identify transcriptional changes associated with the progression of Alzheimer’s disease (AD) pathology. Their data support an immune-response component in the accumulation of tau and reveal molecular pathways associated with AD neuropathology.

## Introduction

Alzheimer’s disease (AD) is a chronic neurodegenerative disorder that is characterized by progressive neuropathology and associated cognitive and functional decline (Scheltens et al., 2016). In addition to the loss of synapses and neurons (manifesting as brain atrophy), AD involves two neuropathological hallmarks: (1) the formation of neurofibrillary tangles (NFTs) that result from the intracellular aggregation of hyperphosphorylated tau protein, also a characteristic of other neurodegenerative disorders including frontotemporal dementia (FTD), and (2) the development of amyloid plaques, which are extracellular deposits composed mainly of β-amyloid (Aβ) protein that have been the focus of extensive efforts in drug discovery. Although these neuropathological signatures of AD have been relatively well described in post-mortem human brain tissue, their exact mechanistic role in disease onset and progression remains poorly understood (De Strooper and Karran, 2016). There have been considerable advances in identifying the genetic risk factors for both familial and sporadic forms of AD; in addition to the autosomal-dominant mutations in *APP*, *PSEN1*, and *PSEN2* that cause early-onset familial AD (Guerreiro et al., 2012), the power of genome-wide association studies (GWAS) and exome sequencing in large sample cohorts (Cruchaga et al., 2014, Guerreiro et al., 2013, Hollingworth et al., 2011, Jansen et al., 2019, Jonsson et al., 2013, Lambert et al., 2013, Sims et al., 2017) has been used to identify both common and rare variants associated with late-onset AD. Although the mechanisms by which associated variants mediate disease susceptibility are not well understood, many of the variants are non-coding and hypothesized to involve regulatory disruption to transcriptional networks across affected brain regions.

Mouse models of tau and amyloid have played a major role in defining critical pathology-related processes, including facilitating our understanding of the brain’s transcriptional response to the production and gradual deposition of tau and amyloid into tangles and plaques (Götz and Ittner, 2008). Recent studies have identified widespread gene expression differences in transgenic (TG) mice harboring a diverse range of AD-associated mutations (Castillo et al., 2017, Landel et al., 2014, Matarin et al., 2015, Rothman et al., 2018, Swarup et al., 2019, Wes et al., 2014, Neuner et al., 2019). However, most analyses to date have been undertaken on relatively small numbers of animals and have not attempted to directly relate transcriptional alterations to the progressive burden of pathology from multiple brain regions in the same mice.

In this study, we systematically assess transcriptional signatures associated with the progression of AD pathology in the mouse brain, using highly parallel RNA sequencing (RNA-seq) to quantify gene expression changes in the entorhinal cortex, a region defined by primary and early neuropathology in human AD (Braak and Braak, 1991). We used well-characterized TG mouse models of both tau and amyloid pathology, collecting transcriptional data at multiple time points carefully selected to span from early to late stages of neuropathology in each model (see Figure S1). First, to investigate transcriptional signatures of progressive tau pathology, we used the rTg4510 mouse model, which overexpresses a human mutant (P301L) form of the microtubule-associated protein tau (MAPT) (Ramsden et al., 2005, Santacruz et al., 2005). Second, to investigate amyloid pathology, we used the J20 mouse line, which expresses a mutant (K670N/M671L and V717F) form of the human amyloid precursor protein (APP) (Hsia et al., 1999, Mucke et al., 2000). Specific differentially expressed genes were validated using qPCR and subsequently tested in the hippocampus, another brain region affected by early pathology. Transcriptional profiles were related to detailed neuropathological measurements of tau and amyloid burden in the same mice, enabling us to directly relate expression changes to the progressive accumulation of neuropathology. We identified robust genotype-associated differences in entorhinal cortex gene expression in both models and widespread transcriptional changes paralleling the development of tau pathology in rTg4510 mice. Systems-level analyses uncovered discrete co-expression networks associated with the progression of tau pathology that were enriched for genes and pathways implicated in the onset of AD. Finally, we compared these networks with those identified in AD patients, finding considerable overlap with disease-associated co-expression modules identified in the human cortex.

## Results

### TG Mice with APP and MAPT Mutations Are Characterized by Progressive Neuropathology

**Figure 1:**
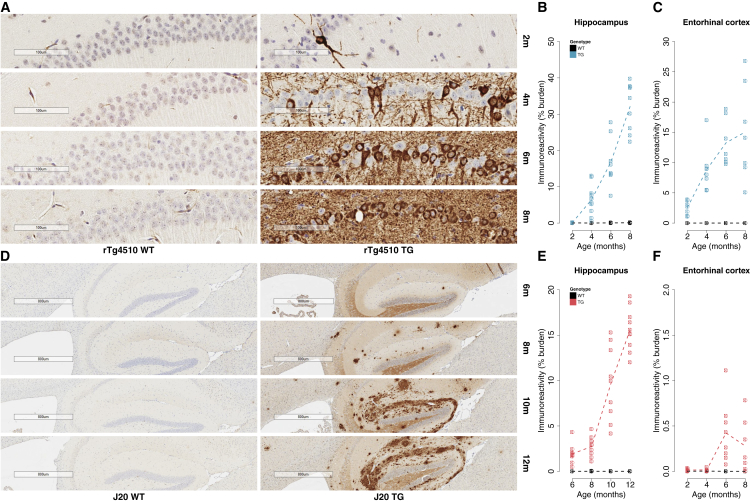
Transgenic Models Expressing Mutant Human *MAPT* and *APP* Exhibit Progressive Neuropathology (A) Representative immunohistochemistry images from the hippocampus (CA1) showing accumulation of tau pathology in rTg4510 transgenic (TG) mice compared with wild-type (WT) control mice at 2, 4, 6, and 8 months of age. (B) There was a progressive increase in hippocampal tau in rTg4510 TG but not WT animals (total n = 73 animals; 8–10 animals per group; factorial ANOVA, F[3, 66] = 69.76, p = 1.96E−20). (C) There was a progressive increase in tau in the entorhinal cortex in rTg4510 TG but not WT animals (total n = 59 animals; 6–8 animals per group; factorial ANOVA, F[3, 51] = 9.86, p = 3.10E−5). (D) Representative immunohistochemistry images from the hippocampus showing progressive accumulation of amyloid pathology in J20 TG mice compared with WT mice at 6, 8, 10, and 12 months of age. (E) There was a progressive increase in hippocampal amyloid in J20 TG but not WT mice (total n = 77 animals; 9 or 10 animals per group; factorial ANOVA, F[3, 68] = 66.85, p = 3.00E−20). (F) There was a modest increase in amyloid in the entorhinal cortex of J20 TG but not WT mice, particularly in later stages (total n = 62 animals; 7 or 8 animals per group; factorial ANOVA, F[3, 53] = 6.42, p = 0.00086). Dashed lines represent mean paths of pathological burden across the four age groups.

In both models, right brain hemisphere tissue sections from TG and wild-type (WT) littermate control female mice (STAR Methods) were used to quantify the progression of neuropathology across multiple brain regions (Figures S2A and S2N). First, in rTg4510 mice we measured levels of phosphorylated tau at 2, 4, 6, and 8 months, comparing them with WT controls at the same ages (n = 7–10 animals per group, total n = 74). We identified a dramatic accumulation of tau pathology in both the hippocampus (factorial ANOVA, F[3, 66] = 69.76, p = 1.96E−20) and entorhinal cortex (factorial ANOVA, F[3, 51] = 9.86, p = 3.10E−5) (Figures 1A–1C). Highly significant increases in phosphorylated tau were also observed within specific sub-regions of the hippocampus and each of the additional cortical regions we quantified (Figures S2B–S2M). Previous studies have shown that rTg4510 mice develop pretangles around 2.5 months of age, with NFT pathology starting in the neocortex and progressing rapidly into the hippocampus and limbic structures with increasing age (Ramsden et al., 2005, Sahara et al., 2014, Santacruz et al., 2005). The spread of tau pathology in rTg4510 mice therefore reflects the spread of NFTs with increasing Braak stage in AD (Braak and Braak, 1991). Second, we quantified levels of amyloid pathology in J20 mice at ages 6, 8, 10, and 12 months, comparing them with WT controls at the same ages (n = 9 or 10 animals per group, total n = 73). We again identified dramatic increases in pathology in the hippocampus (factorial ANOVA, F[3, 68] = 66.85, p = 3.00E−20), with a more modest increase in the entorhinal cortex (factorial ANOVA, F[3, 53] = 6.42, p = 0.00086) (Figures 1D–1F), and a significant accumulation of amyloid was also observed in each of the other cortical regions examined (Figures S2O–S2R). To our knowledge, no previous study has quantified amyloid progression in entorhinal cortex in J20 mice, and it is noteworthy that we observe lower levels of pathology in this region compared with the hippocampus and other neocortical regions. Our results concur with previous data highlighting progressive deposition of amyloid plaques in the hippocampus and neocortex of J20 mice at 5–7 months and ubiquitous plaque pathology by 8–10 months of age (Harris et al., 2010a, Mucke et al., 2000), reflecting the progressive deposition of amyloid seen in individuals with AD (Thal et al., 2002). Finally, we quantified neuropathology in the thalamus from both models, which, as expected, showed markedly lower levels of tau (rTg4150; Figure S2M) and amyloid (J20; Figure S2R) pathology relative to the other brain regions tested.

### The rTg4510 Model of Tau Pathology Is Characterized by Widespread Transcriptional Differences in the Entorhinal Cortex

The entorhinal cortex was dissected from the left hemisphere of the brain from each individual mouse (TG and WT) at each of the four time points. High-quality RNA (mean RNA integrity number [RIN] rTg4510 = 8.9 [SD = 0.2], mean RIN J20 = 8.6 [SD = 0.3]) was isolated from each sample (total n = 121) and used for highly parallel RNA-seq (STAR Methods). After stringent quality control (QC) of the raw RNA-seq data (STAR Methods), we obtained a mean of 18.18 million (SD = 3.33 million) sequencing reads per sample for the rTg4510 dataset and a mean of 22.05 million (SD = 2.88 million) sequencing reads per sample for the J20 dataset (Table S1), with no difference in read depth between TG and WT controls (rTg4510: two-tailed unpaired t test, t[57] = 1.35, p = 0.18; J20: two-tailed unpaired t test, t[60] = 0.41, p = 0.18). We quantified read counts for each transcript and evaluated differences in gene expression between TG and WT animals for each model (STAR Methods). Our extensive gene expression dataset generated on rodent models of AD pathology is well powered to identify transcriptional variation associated with mutations in *MAPT* and *APP* and the progressive changes in gene expression accompanying the development of AD pathology in TG mice (Figure S1C).

**Figure 2:**
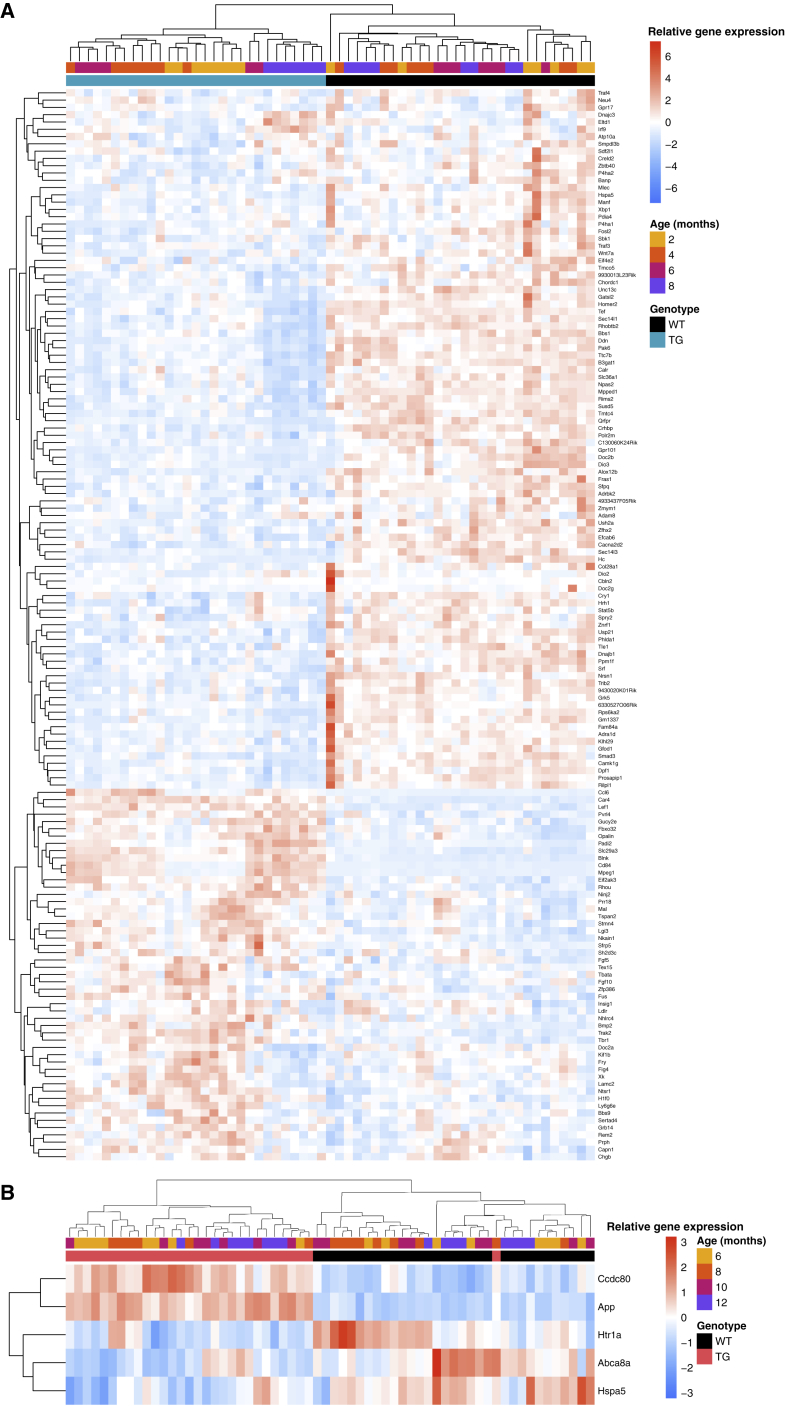
Genotype-Associated Transcriptional Variation Robustly Discriminates between Transgenic and Wild-Type Mice Normalized DESeq2 read counts, relative to mean levels of expression across all individual mice, are represented in the heatmaps (scaled) from high (red) to low (blue). (A) Hierarchical clustering of individual mice on the basis of expression levels for differentially expressed genes associated with rTg4510 genotype (n = 59 mice [29 TG, 30 WT], 147 transcripts). (B) Hierarchical clustering of individual mice on the basis of expression levels for differentially expressed genes associated with J20 genotype (n = 62 mice [30 TG, 32 WT], 5 transcripts).

Across all samples, extensive differences in gene expression were identified in rTg4510 TG animals relative to WT control mice (n = 29 TG, n = 30 WT); gene expression results for all 18,822 detected transcripts are available for download (STAR Methods). In total we identified 154 differentially expressed transcripts at a stringent false discovery rate (FDR) of <0.05 (Figure 2A; Table S2). Among these, there was a significant (exact binomial test, n = 154 transcripts, p = 0.00014) enrichment of downregulated transcripts (n = 101 transcripts [66%] with reduced expression in TG compared with n = 53 transcripts [34%] with elevated expression in TG). Of note, differences for five of these transcripts are likely to reflect known deletions of the transgene integration sites for the CaMKIIα-tTA (encompassing *Wdr60*, *Esyt2*, *Ncapg2*, and *Ptprn2*) and MAPT (encompassing *Fgf14*) transgenes (Goodwin et al., 2017, Gamache et al., 2019). Given the high homology between transcribed regions of the human and mouse tau gene, we also find elevated levels of *Mapt* (Wald statistic = 11.11, log_2_ fold change = 0.50, FDR = 7.08E−25) (Figure S3A), confirming stable activation of the *MAPT* transgene in TG mice; of note, human-specific *MAPT* sequence domains were detected only in TG mice (Figures S3B and S3C). Furthermore, because the rTg4510 transgene is inserted into the context of two untranslated exons of the mouse prion protein gene (*Prnp*), as expected we observe elevated expression of these *Prnp* domains in TG mice (Wald statistic = 25.40, log_2_ fold change = 1.54, FDR = 4.88E−138).

Beyond these expected direct transgene-induced changes, we observed evidence for widespread transcriptional consequences of the rTg4510 genotype (Table S2). The most significant rTg4510-associated differentially expressed transcript is *Car4*, which encodes carbonic anhydrase 4 (upregulated in TG mice; Wald statistic = 8.36, log_2_ fold change = 1.11, FDR = 2.41E−13). Other differentially expressed genes in mice carrying the rTg4510 transgene include *Gpr17*, which encodes G protein-coupled receptor 17, which is involved in regulating oligodendrocyte differentiation and maturation (Chen et al., 2009) (downregulated in TG mice; Wald statistic = −6.73, log_2_ fold change = −0.62, FDR = 5.11E−08); *Blnk*, which encodes a cytoplasmic linker protein that plays a critical role in B cell development and is involved in the TREM2 activation pathway (Zajkowicz et al., 2018) (upregulated in TG mice; Wald statistic = 6.48, log_2_ fold change = 0.80, FDR = 2.12E−07); and *Hspa5* (also known as *Bip* or *Grp78*), which encodes a member of the heat shock protein 70 (HSP70) family that is localized in the lumen of the endoplasmic reticulum (ER) and involved in the folding and assembly of proteins and has been previously implicated in neuroprotection and AD (Casas, 2017, Hoozemans et al., 2005) (downregulated in TG mice; Wald statistic = −6.16, log_2_ fold change = −0.58, FDR = 1.37E−06). Hierarchical clustering of individual mice on the basis of expression levels for genotype-associated transcripts robustly discriminates between rTg4510 and WT groups (Figure 2A). Within the rTg4510 TG group, samples also cluster by time point, suggesting, importantly, that there are progressive changes in gene expression within the mutant mice and highlighting the value of performing longitudinal analyses. Finally, using qPCR, we validated top-ranked genotype-associated expression differences in the entorhinal cortex (Figure S4; Table S3) and found parallel dysregulation of these genes in matched hippocampus samples dissected from the same individuals (*Car4*: t test statistic = −29.05, FDR = 4.90E−32; *Gpr17*: t test statistic = −2.19, FDR = 0.035; *Blnk*: t test statistic = −18.13, FDR = 3.98E−23; *Hspa5*: t test statistic = 4.14, FDR = 0.00019) (Figure S4; Table S3).

### The J20 Model of Amyloid Pathology Is Characterized by Differential Expression of *Ccdc80*, *Abca8a*, *Htr1a*, and *Hspa5*

Relative to the widespread transcriptional signatures associated with the rTg4510 model, fewer significant expression differences were identified in J20 TG mice compared with WT control mice (n = 30 TG, n = 32 WT); gene expression results for all 18,745 expressed transcripts are available for download (STAR Methods). As expected, there was an apparent upregulation of *App* (Wald statistic = 8.55, log_2_ fold change = 0.66, FDR = 2.37E−13) (Figure S3D), reflecting the high sequence homology with the human *APP* transgene and confirming stable activation of the mutant transgene in TG mice; of note, we mapped our RNA-seq reads to human-specific APP sequence domains and observed signal only in TG animals (Figures S3E and S3F). In total we identified four additional differentially expressed transcripts at FDR < 0.05 (Figure 2B; Table S2): *Ccdc80*, encoding a protein involved in cell adhesion and matrix assembly (O’Leary et al., 2013) (upregulated in TG samples; Wald statistic = 6.37, log_2_ fold change = 0.81, FDR = 1.74E−06); *Abca8a*, encoding a member of the A-subclass of ATP-binding cassette (ABC) transporter family that regulates brain lipid homeostasis and has been implicated in AD (Piehler et al., 2012) (downregulated in TG samples; Wald statistic = −4.67, log_2_ fold change = −0.81, FDR = 0.02); *Htr1a*, encoding a major G protein-coupled serotonin receptor, the 5-HT_1A_ receptor, which is widely expressed in the central nervous system (CNS) (downregulated in TG samples; Wald statistic = −4.48, log_2_ fold change = −0.51, FDR = 0.035); and *Hspa5* (downregulated in TG samples, Wald statistic = −4.36, log_2_ fold change = −0.28, FDR = 0.049). Overall, expression of these genotype-associated transcripts discriminates between J20 and WT groups (Figure 2B), although, in contrast to the rTg5410 differentially expressed transcripts, there are no clear age effects in the J20 TG mice. Although the transcriptional changes associated with the rTg4510 and J20 genotypes are generally distinct—there is no robust correlation of effect sizes (TG versus WT) between models for differentially expressed transcripts identified in either the rTg4510 (Pearson correlation, r = 0.15, p = 0.063; Figure S5A) or J20 (r = 0.66, p = 0.23; Figure S5B) models—it is noteworthy that *Hspa5* is significantly downregulated (FDR < 0.05) in the entorhinal cortex in the same direction in both models, implicating a role for ER stress in both tau and amyloid pathology. Of note, recent evidence suggests that activation of this gene is essential for the initiation of the unfolded protein response (UPR) in Parkinson’s disease (Enogieru et al., 2019). Finally, using qPCR we confirmed differential expression in the entorhinal cortex and found parallel dysregulation of two of the J20-associated transcripts in the hippocampus dissected from the same individuals (*Abca8a*: t test statistic = 2.74, FDR = 0.015; *Ccdc80*: t test statistic = −8.57, FDR = 7.60E−11) (Figures S4I–S4L; Table S3).

### Progressive Changes in Gene Expression Mirror the Development of Neuropathology in the rTg4510 Model of Tau Pathology

**Figure 3:**
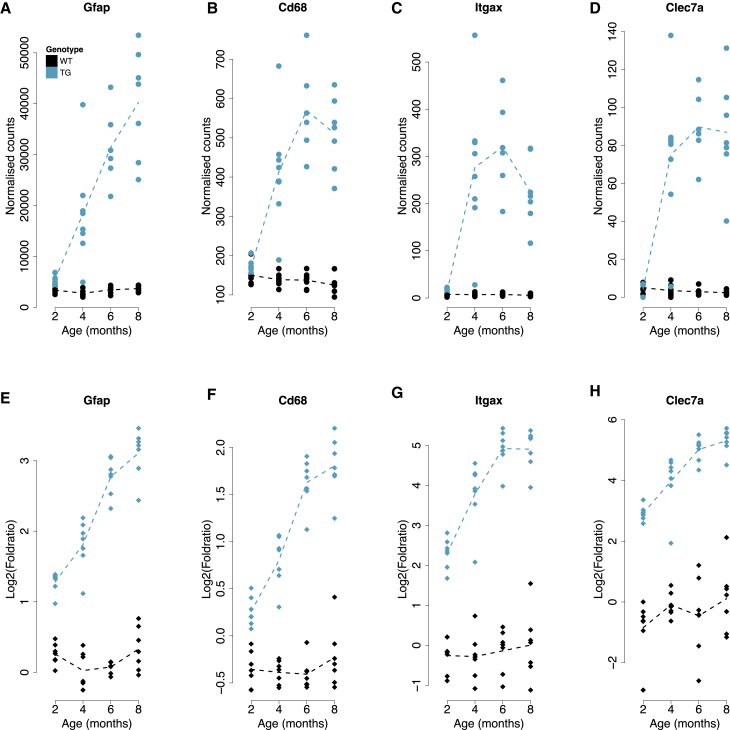
Top-Ranked Progressively Differentially Expressed Genes in rTg4510 Mice Top row shows normalized RNA-seq read counts for entorhinal cortex. Bottom row shows targeted qPCR data for the same genes in matched hippocampus tissue. Dashed lines represent mean paths for each time point. (A) *Gfap* (LRT statistic = 106.32, log_2_ fold change [2–8 months] = 2.75, FDR = 1.28E−18). (B) *Cd68* (LRT statistic = 103.77, log_2_ fold change [2–8 months] = 1.85, FDR = 2.26E−18). (C) *Itgax* (LRT statistic = 86.85, log_2_ fold change [2–8 months] = 4.42, FDR = 6.54E−15). (D) *Clec7a* (LRT statistic = 83.20, log_2_ fold change [2–8 months] = 5.37, FDR = 2.97E−14). (E) *Gfap* (interaction F statistic = 36.93, FDR = 3.69E−11). (F) *Cd68* (interaction F statistic = 30.86, FDR = 3.33E−10). (G) *Itgax* (interaction F statistic = 12.94, FDR = 9.80E−6). (H) *Clec7a* (interaction F statistic = 3.74, FDR = 0.034).

Given the progressive accumulation of brain neuropathology in TG mice (Figure 1), we next explored temporal changes in gene expression associated with genotype to identify transcriptional signatures paralleling the increases in tau and amyloid pathology in TG mice over time (Figure S1C). We initially focused on the rTg4510 mice given the clear temporal clustering of samples among genotype-associated differentially expressed transcripts identified in this model (Figure 2A). First, using an approach designed to identify interactions between genotype (TG versus WT) and age group, we identified 1,762 transcripts (FDR < 0.05) whose expression significantly changed with time in TG rTg4510 mice (Table S4). The top progressively altered gene in rTg4510 TG mice was *Gfap*, encoding glial fibrillary acidic protein (GFAP), a gene predominantly expressed in both mouse and human astrocytes (Raff et al., 1979, Zhang et al., 2016) and known to be upregulated in reactive astrocytes associated with brain pathology (Ben Haim et al., 2015). GFAP is extremely sensitive to changes in the homeostasis of the brain and responds early in the course of neurodegenerative disease (Sofroniew, 2009). We found that *Gfap* was dramatically upregulated across age groups (Figure 3A; likelihood ratio test [LRT] statistic = 106.321, FDR = 1.28E−18), similar to results from another study reporting age-dependent (12–18 months) upregulation of hippocampal *Gfap* in tau (*CaMKII*-*MAPT* P301L) and amyloid (*APP*/*PSEN1*) mouse models (Matarin et al., 2015) and paralleling the astrogliosis observed in human AD brain (Liddelow et al., 2017, Panter et al., 1985). Other top-ranked genes progressively altered in rTg4510 mice were notably enriched for microglial markers previously shown to be upregulated in AD (Hopperton et al., 2018, Keren-Shaul et al., 2017), including *Cd68* (Figure 3B; LRT statistic = 103.77, FDR = 2.26E−18), *Itgax* (or *Cd11c*) (Figure 3C; LRT statistic = 86.85, FDR = 6.54E−15), and *Clec7a* (Figure 3D; LRT statistic = 83.20, FDR = 2.97E−14). These genes have been previously reported to be upregulated in hippocampal tissue from 6-month-old rTg4510 female mice (Wes et al., 2014), in isolated microglia from rTg4510 mice (Wang et al., 2018), in the cortex of amyloid mice at late stages of pathology (Rothman et al., 2018), and in the neocortex, hippocampus, and microglia of mice with amyloid and tau pathology (Keren-Shaul et al., 2017, Landel et al., 2014). Of note, the list of transcripts progressively altered in rTg4510 mice also includes genes robustly associated with familial AD from genetic studies of human patients, including *App* (Figure S6A; LRT statistic = 13.88, FDR = 0.037), a key driver of amyloid pathology, and genes annotated to both common and rare variants identified in GWAS and exome-sequencing studies of late-onset sporadic AD (LOAD), including *Trem2* (LRT statistic = 43.82, FDR = 3.73E−07), *Pld3* (LRT statistic = 36.80, FDR = 5.80E−06), *Frmd4a* (LRT statistic = 27.81, FDR = 0.00022), *Clu* (LRT statistic = 27.73, FDR = 0.00023), *Apoe* (LRT statistic = 22.99, FDR = 0.0014), *Picalm* (LRT statistic = 21.37, FDR = 0.0025), *Cd33* (LRT statistic = 27.32, FDR = 0.00026), and *Abi3* (LRT statistic = 17.10, FDR = 0.012) (Figures S6B–S6I).

**Figure 4:**
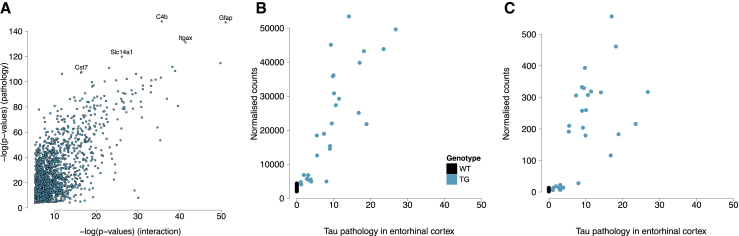
Transcriptional Variation Associated with Levels of Tau Pathology in rTg4510 Entorhinal Cortex (A) Association statistics for transcripts differentially expressed with age in rTg4510 TG mice were highly correlated with those from analyses of tau pathology in the entorhinal cortex (r = 0.70, p < 1E−200). (B and C) There was a notable overlap in top-ranked genes, including (B) *Gfap* (r = 0.89, p = 4.60E−20) and (C) *Itgax* (r = 0.80, p = 1.41E−13).

Given that there is some variability in gene expression and tau pathology within each of the four TG age groups, we next related RNA-seq to immunohistochemistry data (STAR Methods) to identify transcripts whose expression is significantly associated with actual levels of tau quantified in the entorhinal cortex and hippocampus (Table S5) from in the same individual mice. As expected, association statistics for transcripts differentially expressed with age in TG mice were highly correlated with those from analyses of tau pathology in both brain regions (entorhinal cortex: r = 0.70, p < 1E−200 [Figure 4A]; hippocampus: r = 0.58, p = 1.8E−149 [Figure S5D]), with a notable overlap in top-ranked genes including *Gfap* (Wald statistic = 16.97, FDR = 1.32E−60; Figure 4B) and *Itgax* (Wald statistic = 16.01, FDR = 6.89E−54; Figure 4C). We used qPCR to (1) validate top-ranked temporal and pathology-associated changes in *Gfap*, *Cd68*, *Itgax*, and *Clec7a* expression; (2) confirm changes in levels of selected genes also implicated by genetic studies of AD (*Apoe*, *Cd33*, *Clu*, *Frmd4a*, *Picalm*, *Pld3*, and *Trem2*) (FDR < 0.05 for all; Table S3); and (3) identify parallel changes in the expression of the majority of these genes in the hippocampus from the same individual mice (*Gfap*: interaction F statistic = 36.93, FDR = 3.69E−11; *Cd68*: interaction F statistic = 30.86, FDR = 3.33E−10; *Itgax*: interaction F statistic = 12.94, FDR = 9.80E−6; *Clec7a*: interaction F statistic = 3.74, FDR = 0.034) (Figures 3E–3H; Table S3).

Overall, the progression of tau pathology is associated with the downregulation of key neuronal markers, with an upregulation of transcripts associated with astrocytes and microglia (Figure S7). We used GOseq (STAR Methods) to identify ontological enrichments among genes characterized by progressively altered gene expression in rTg4510 mice, finding highly significant enrichments for immune related biological pathways including “immune system process” (FDR = 1.03E−25), “defence response” (FDR = 2.98E−24), and “immune response” (FDR = 4.79E−24) (Table S6). Given these findings, we next quantified Iba1, a microglia/macrophage-specific calcium-binding protein (Ohsawa et al., 2004), in matched tissue sections from the right brain hemisphere (n = 7–10 animals per group, total n = 70), observing a significant increase in all brain regions (hippocampus: factorial ANOVA, F[3, 62] = 12.60, p = 1.56E−06; cortex: factorial ANOVA, F[3, 62] = 18.13, p = 1.47E−08; thalamus: factorial ANOVA, F[3, 62] = 18.85, p = 8.37E−09) (Figures S2K–S2M). Together our results reflect the dramatic upregulation of microglial genes observed in studies of other AD rodent models (Kamphuis et al., 2016, Kan et al., 2015, Keren-Shaul et al., 2017, Landel et al., 2014, Matarin et al., 2015, Rothman et al., 2018), and support a role, either causal or consequential, for dysregulation of the CNS immune processes in the development of AD pathology. Of note, recent transcriptional studies in human brain have shown that microglial gene networks are upregulated in response to AD neuropathology (Felsky et al., 2019).

### In Contrast to the Dramatic and Progressive Transcriptional Changes Identified in rTg4510 Mice, Relatively Few Changes Were Associated with the Development of Pathology in J20 Mice

**Figure 5:**
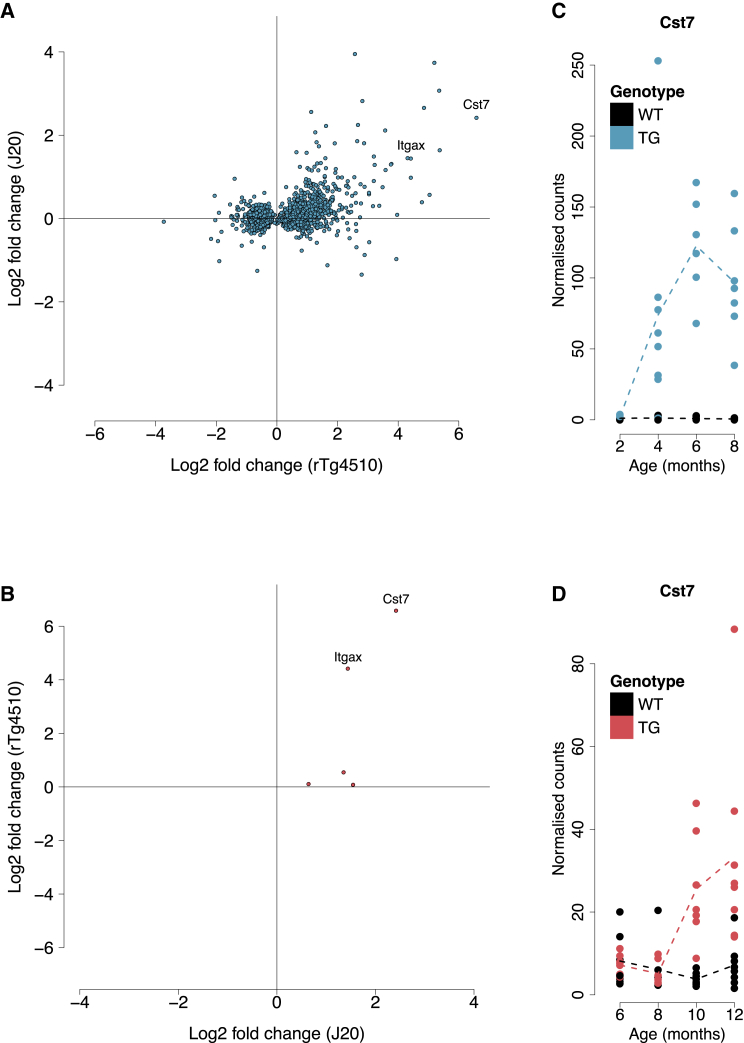
Effect Sizes for Differentially Expressed Transcripts Associated with Progression of Tau in rTg4510 Mice Are Correlated with Those Observed with Progression of Amyloid in J20 Mice (A) Positive correlation for effect size (log_2_ fold change from latest time point compared with baseline) for significant transcripts in rTg4510 mice (r = 0.46, p = 1.50E−92; exact binomial test, n = 1,762 transcripts, p = 1.97E−05). (B) Two transcripts (*Cst7* and *Itgax*) were significantly associated with the progression of both tau (rTg4510) and amyloid (J20) pathology (r = 0.77, p = 0.13; exact binomial test, n = 5 transcripts, p = 0.13). (C) *Cst7* gene expression in rTg4510 mice (total n = 59 animals, 6–8 animals per group, LRT statistic = 36.10, log_2_ fold change [2–8 months] = 6.59, FDR = 7.71E−06). rTg4510 transgenic (TG) female mice compared with wild-type (WT) littermate control mice. (D) *Cst7* gene expression in the J20 mice (total n = 62 animals, 6–8 animals per group, LRT statistic = 37.37, log_2_ fold change [6–12 months] = 2.42, FDR = 0.00072). J20 transgenic (TG) female mice compared with wild-type (WT) littermate control mice. *Itgax* gene expression in rTg4510 and J20 mice is shown in Figure 3C and Figure S8, respectively.

In total we identified five transcripts (*Cst7*, *Wdfy1*, *Grxcr2*, *Itgax*, and *Ifitm1*) whose expression profiles significantly changed (FDR < 0.05) with age in TG J20 mice (Table S4). Using our immunohistochemistry data, we identified 11 transcripts significantly associated with levels of amyloid pathology in the entorhinal cortex and 223 associated with hippocampal pathology (Table S5). Although pathology association statistics for common differentially expressed genes were highly correlated across both brain regions (Pearson correlation, r = 0.87, p = 0.0011), the higher number of associations identified for the hippocampus likely reflects the more abundant pathology observed in this brain region in TG mice (Figures 1E and 1F). The relatively small overall number of significantly altered genes in J20 mice potentially reflects the slower and later accumulation of pathology in these mice compared with rTg4510 mice. Previous work has also shown relatively limited amyloid pathology in J20 entorhinal cortex even at 14 months of age (Harris et al., 2010b), with neuronal cell loss varying by brain region (Wright et al., 2013). Nevertheless, we found that effect sizes for the transcripts identified as being progressively dysregulated in rTg4510 and in J20 mice were significantly correlated across both models, suggesting some common molecular signals associated with both tau and amyloid pathology (Figure 5). Interestingly, two genes (*Cst7* and *Itgax*) identified as being associated with progressive tau pathology in rTg4510 mice were also significantly associated with amyloid pathology in J20 mice (Figure 5; Figure S8). Like *Itgax*, *Cst7* has been shown to be a marker for activated microglia and upregulated in AD pathology (Keren-Shaul et al., 2017, Ofengeim et al., 2017); of note, *Cst7* was previously reported to be the top upregulated gene in cortex samples from 12-month-old APP NL-G-F knockin mice (Castillo et al., 2017). Finally, we used qPCR to confirm significant temporal and pathology-associated changes in expression of *Cst7* and *Itgax* in the J20 entorhinal cortex (Table S3) and identified parallel changes in the hippocampus from the same individuals (*Cst7*: interaction F statistic = 48.16, FDR = 3.76E−13; *Itgax*: interaction F statistic = 13.09, FDR = 1.31E−5) (Table S3). Of note, the changes identified in the hippocampus for these genes were considerably larger than those in the entorhinal cortex, further reflecting the higher hippocampal levels of amyloid pathology.

### Transcriptional Changes Identified in rTg4510 Mice Reflect Those Observed in Other Models of Tau Pathology

A number of recent studies have also described evidence for differential gene expression in TG models of familial AD gene mutations (Castillo et al., 2017, Keren-Shaul et al., 2017, Landel et al., 2014, Matarin et al., 2015, Rothman et al., 2018, Wang et al., 2018, Wes et al., 2014). We therefore explored hippocampal RNA-seq data from two other TG models (TAU [CaMKII-MAPTP301L] and TAS10 [SwAPP, K670N/M671L]) (Matarin et al., 2015, Salih et al., 2019) to identify consistencies in the transcriptional signatures between different models of tau and amyloid pathology (STAR Methods; http://www.mouseac.org). Effect sizes for rTg4510 genotype-associated transcripts also present in the Mouseac TAU RNA-seq dataset (n = 138) were significantly correlated between the two models (Pearson correlation, r = 0.33, p = 7.7E−05). Despite this consistency in effect sizes, many of the differentially expressed genes associated with rTg4510 genotype were not statistically replicated in the TAU model (Figure S5C; Table S7), although this likely reflects the distinct genetic background of the different TG lines and the modest power to detect effects given the small number of samples from the Mouseac dataset (n = 49 RNA-seq samples, one to four animals per age group, after filtering for samples with complete phenotypic data). As expected, given the limited evidence for consistency in genotype effects between rTg4510 and J20 mice, there was no correlation between effects observed in rTg4510 and TAS10 mice for the 145 rTg4510-associated genes present in both datasets. In contrast, association statistics for the 1,640 transcripts identified as being progressively altered with age in rTg4510 mice and also present in the Mouseac datasets (Table S7) were significantly correlated with those for the same genes in both TAU (r = 0.46, p = 1.2E−86) and TAS10 (r = 0.23, p = 3.9E−21) TG mice (Figures S5E and S5F). Given the small number of progressive alterations observed in J20 mice, it was not possible to systematically explore overlaps between differentially regulated genes in this model and the two Mouseac models. Of note, however, the two genes identified as being temporally altered in both rTg4510 and J20 mice, *Cst7* and *Itgax*, were both similarly altered in both the TAU and TAS10 models (Figure S8).

### Gene Co-expression Networks Associated with the Progression of Tau Pathology Are Enriched for Functional Pathways Related to Synaptic Transmission, the Immune System, and Glial Cell Activation

**Figure 6:**
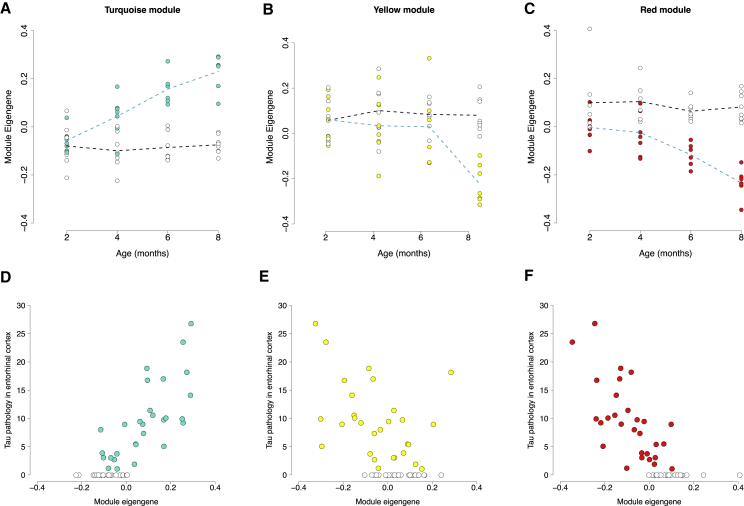
Variation in Entorhinal Cortex Co-expression Modules Parallels the Accumulation of Tau Pathology in rTg4510 Mice (A–C) Shown are module eigengene values for individual mice at four time-points for (A) the turquoise module (n = 3,091 transcripts; linear regression, F[3, 50] = 12.18, β = 0.28, p = 4.23E−06); (B) the yellow module (n = 1,102 transcripts; linear regression, F[3, 50] = 5.79, β = −0.29, p = 0.0018); and (C) the red module (n = 726 transcripts; linear regression, F[3, 50] = 5.58, β = −0.21, p = 0.0022). The same modules are correlated with tau pathology in entorhinal cortex in the same individuals. (D) Positive correlation between module eigengene in the turquoise module and tau pathology in the entorhinal cortex (Pearson correlation, r = 0.82, p = 4.00E−15). (E) Negative correlation between module eigengene in the yellow module and tau pathology in the entorhinal cortex (Pearson correlation, r = −0.50, p = 7.52E−5). (F) Negative correlation between module eigengene in the red module and tau pathology in the entorhinal cortex (Pearson correlation, r = −0.75, p = 8.01E−12). Total n = 58 animals (6–8 animals per group). Colored circles represent rTg4510 TG mice, and white circles represent WT control mice. Dashed lines represent mean paths for each time point.

Given the dramatic transcriptional changes identified in rTg4510 mice, we next used weighted gene correlation network analysis (WGCNA) (STAR Methods) to identify discrete co-expression modules and describe systems-level transcriptional variation associated with rTg4510 genotype and progression of tau pathology. We constructed co-expression networks using entorhinal cortex RNA-seq data from rTg4510 TG and WT mice (n = 58 mice), identifying 18 discrete co-expression modules (Figure S9). Next, we used a linear regression model (STAR Methods) and identified six co-expression modules (here named “salmon,” “turquoise,” “purple,” “yellow,” “light-cyan,” and “red”) that were significantly (Bonferroni-corrected p < 0.0028) associated with rTg4510 genotype (Figures S9A and S9B; Table S8). These tau-associated co-expression modules are highly enriched for molecular functions and biological pathways directly related to AD: the red module, which was downregulated in TG mice compared with WT mice (β = −0.18, p = 1.43E−10), is highly enriched for functional pathways involved in synaptic transmission; the turquoise module, which was upregulated in TG mice compared with WT mice (β = 0.18, p = 3.04E−10), is enriched for pathways involved in activation of the immune system; the salmon module, which was consistently upregulated in TG mice compared with WT mice (β = 0.14, p = 3.58E−06), is enriched for genes involved in myelination and glial cell activation; the purple module, which was downregulated in TG mice compared with WT mice (β = −0.13, p = 0.00012), is enriched for pathways related to cellular component disassembly; and the yellow module, which was downregulated in TG mice compared with WT mice (β = −0.10, p = 0.0015), is enriched for pathways related to mitochondria and synaptic processes (Table S6). The module eigengenes for three of these co-expression modules (turquoise, yellow, and red) exhibited a significant interaction between genotype and age in rTg4510 mice (Figure S9A; Table S8), suggesting that they are temporally linked to the development of tau pathology in TG mice (Figures 6A–6C). The turquoise module becomes increasingly upregulated with time in TG mice (β = 0.28, p = 4.23E−06), the red module becomes increasingly downregulated with time in TG mice (β = −0.21, p = 0.0022), and the yellow module becomes downregulated specifically during the later time points in TG mice (β = −0.29, p = 0.0018). Using the matched immunohistochemistry data generated across multiple brain regions for each mouse, we were able to explore the relationship between co-expression modules and actual tau pathology in rTg4510 mice, confirming that the turquoise, yellow, and red modules are robustly associated with the accumulation of tau across the brain (Figure S9C). The association with pathology was particularly strong in highly affected brain regions, including the entorhinal cortex (Figures 6D–6F) and hippocampus (Figure S10); in both regions the module eigengene for the turquoise module is positively correlated with levels of tau in TG mice (hippocampus: r = 0.85, p = 1.20E−16; entorhinal cortex: r = 0.82, p = 4.00E−15), and those for the yellow (hippocampus: r = −0.63, p = 2.00E−07; entorhinal cortex: r = −0.50, p = 7.52E−5) and red (hippocampus: r = −0.79, p = 4.61E−13; entorhinal cortex: r = −0.75, p = 8.01E−12) modules are negatively correlated with levels of tau in TG mice. Although these co-expression modules were also correlated with measures of tau pathology in the thalamus, the magnitude of effects was much lower, reflecting the later and less aggressive accumulation of tau in this region of the brain (Figures S10J–S10L).

**Figure 7:**
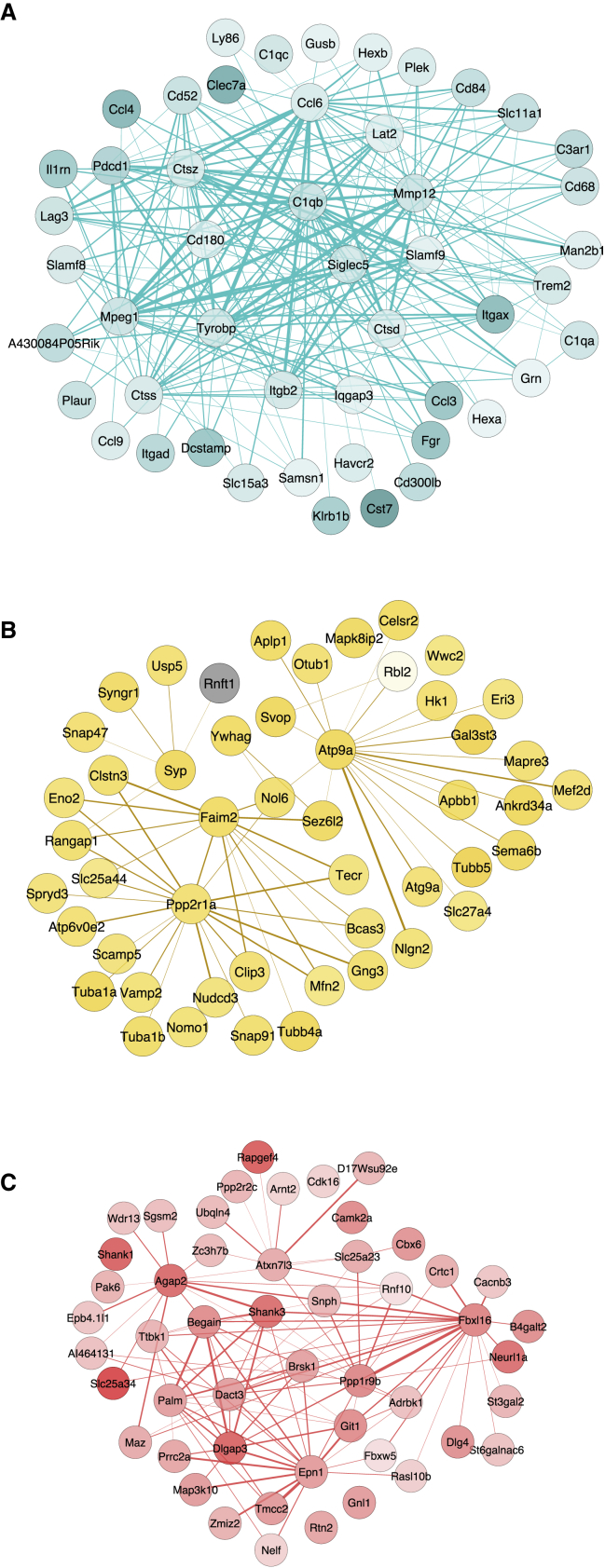
Network Plots Highlighting Core Members of Gene Co-expression Modules Associated with the Development of Tau Pathology Shown are the top 50 nodes (i.e., genes) with the strongest edges (representing individual connections with other genes) for each module. (A) Turquoise module (all upregulated genes). (B) Yellow module (downregulated genes in yellow, upregulated genes in gray). (C) Red module (all downregulated genes). Stronger colors reflect higher absolute log_2_ fold change (8 months against 2 months). Total n = 58 animals (6–8 animals per group).

Within each of these three modules we ranked transcripts on the basis of their intramodular connectivity to identify “hub” genes within each network, finding many genes known to play a major role in the neuro-immunological and neurodegenerative processes involved in AD. In the turquoise module the four genes with the highest intramodular connectivity (i.e., those with most connections to other genes) were *Cd63*, *Msn*, *Npc2*, and *Tnfrsf1a* (Table S9), with other highly interconnected transcripts including several genes identified as having a role in LOAD from GWAS (e.g., *Abca1*, *Clu*, and *Apoe*) in addition to genes previously implicated in AD pathology (e.g., *Itgax*, *Clec7a*, and *Cd68*). Furthermore, genes identified as having the strongest connections (edges) to other genes (nodes) in the turquoise module included *C1qb*, *Mpeg1*, *Tyrobp*, and *Trem2* (Figure 7A). In the yellow module, the four transcripts with the highest intramodular connectivity were *Atp9a*, *Ywhag*, *Rab3a*, and *Svop*, with *App* also being a highly connected gene in this module (Table S9); genes identified as having the strongest connections to other genes included *Atp9a*, *Faim2*, and *Ppp2r1a* (Figure 7B). In the red module, *Atxn7l3*, *Sept5*, *Cbx6*, and *Fbxl16* were the top most connected genes (Table S9); genes with the strongest connections to other genes in the red module included *Dlgap3*, *Shank3*, *Epn1*, and *Fbxl16* (Figure 7C).

### Co-expression Changes Identified in rTg4510 Mice Overlap with AD-Associated Co-expression Changes in Human Brain

We next compared the significant rTg4510 co-expression modules with AD-associated co-expression modules reported in a recent human post-mortem RNA-seq meta-analysis (Logsdon et al., 2019), focusing on modules identified in dorsolateral prefrontal cortex (DLPFC) and temporal cortex (TCX). Briefly, we used a hypergeometric test to identify overlaps between the six rTg4510-associated co-expression modules (salmon, turquoise, purple, yellow, light cyan, and red) and four DLPFC and five TCX AD-associated human co-expression modules (Table S10), restricting our analysis to mouse-human homologs (STAR Methods). After controlling for the number of comparisons performed for each of the human brain regions (DLPFC, p < 0.0021; TCX, p < 0.0017), each of the rTg4510-associated modules was found to significantly overlap with at least one AD-associated module in both human cortical regions. For example, genes in the turquoise rTg4510 module (enriched for pathways involved in activation of the immune system; Table S6) were found to overlap significantly with two human DLPFC modules (“DLPFC-blue” and “DLPFC-brown”) and three TCX modules (“TCX-blue,” “TCX-turquoise,” and “TCX-yellow”) associated with AD from Logsdon et al. (2019); for this module, the largest proportion of overlaps in genes were found with the “DLPFC-blue” module (n = 658 genes, 40.82% of the human module gene list, p < 2.2E−16) and the “TCX-turquoise” module (n = 389 genes, 39.1% of the human module gene list, p < 2.2E−16). Interestingly, GOseq analysis highlighted a strong enrichment for immune response processes among the rTg4510 turquoise module genes overlapping with those in both the “DLPFC-blue” module and the “TCX-turquoise” module (Table S6). Reflecting the similarities between these two human cortex modules, the list of overlapping genes includes many of the core hub transcripts identified in the rTg4510 turquoise module for both the “DLPFC-blue” (e.g., *CD63*, *ABCA1*, *CLU*, *APOE*, *ITGAX*, *CLEC7A*, *C1QB*, *TYROBP*, and *TREM2*) and “TCX-turquoise” (e.g., *CD63*, *ITGAX*, *CLEC7A*, *C1QB*, *TYROBP*, and *TREM2*) human modules. Together, these results indicate that the transcriptional networks associated with tau pathology in rTg4510 mice overlap considerably with those identified in human AD cortex and are involved in driving common molecular pathways.

## Discussion

In this study, we identified transcriptional changes in the entorhinal cortex associated with the progression of AD-associated pathology in TG models of both tau (rTg4510) and amyloid (J20) pathology. We found genotype-associated differences in entorhinal cortex gene expression in both models and identified widespread changes in gene expression paralleling the development of tau pathology in rTg4510 mice and reflecting alterations observed in other models of tau pathology. Specific findings were subsequently validated using qPCR and also tested in the hippocampus, with similar patterns observed across both brain regions. Of note, the list of transcripts progressively altered in rTg4510 mice includes genes robustly associated with familial AD from genetic studies of human patients, including *App* which is a key driver of amyloid pathology. It also includes genes annotated to both common and rare variants identified in GWAS and exome-sequencing studies of LOAD. Systems-level analyses identified discrete co-expression networks associated with the progressive accumulation of tau, with these also being enriched for genes and pathways previously implicated in neuroimmune and neurodegenerative processes driving AD pathology. Further support for upregulation of immune system genes in response to tau pathology comes from our finding of increased expression of complement pathway genes including *C1qa*, *C1qb*, and *C1qc*. Finally, we compared these tau-associated networks with those identified in human post-mortem tissue from AD individuals, finding considerable overlap with disease-associated co-expression modules.

Our study represents a systematic analysis of transcriptional variation in mouse models of tau and amyloid pathology, focusing primarily on changes in the entorhinal cortex, a key region of the brain implicated early in the pathogenesis of AD (Braak and Braak, 1991). Compared with previous studies of transcriptional variation in TG mouse models of AD we profiled a relatively large number of samples spanning multiple time points selected to encompass the development of pathology; our study was therefore well powered to identify gene expression differences associated with both genotype and the progression of AD pathology. Furthermore, we implemented a statistical approach that enabled us to detect progressive changes in gene expression across age between the TG and WT samples, not only identifying stable differences induced by the transgene at each time point but also assessing temporal transcriptional changes relative to baseline within mutant mice. Our detailed immunohistochemical analyses also allowed us to directly compare transcriptional variation with actual tau and amyloid pathology quantified using immunohistochemistry in both the entorhinal cortex and the hippocampus from the same individual mice. We performed extensive work to validate key findings using qPCR, confirming pathology-associated changes in the entorhinal cortex and exploring the extent to which they are paralleled in matched hippocampus tissue dissected from the same mice.

Despite these strengths, our study has a number of important limitations that should be considered. First, to minimize the heterogeneity in our analysis, we profiled only female mice. However, a number of sex differences have been previously reported for these models, with females demonstrating elevated and more progressive pathology than males (Blackmore et al., 2017, Yue et al., 2011). Future work should focus on examining the extent to which the transcriptional profiles identified here are consistent between male and female mice. Second, our analysis was performed on bulk entorhinal cortex tissue, comprising a mix of different neural cell types; consequently, changes in the fractional contribution of any given cell type to the total cellular population will contribute to the observed outcomes at each time point. Given the compelling evidence in our data for an enrichment of microglial markers, previously shown to be upregulated in AD (Hopperton et al., 2018, Keren-Shaul et al., 2017), as well as upregulation of canonical markers of astrocytes (Figure S7), future work should focus on identifying changes that occur within these and other brain cell types. Of note, immunocytochemistry analyses of tissue sections from the left-brain hemisphere of these mice revealed a progressive increase in the microglia/macrophage marker Iba1, indicating that our bulk-tissue RNA-seq measurements reflect real underlying cellular changes. In rTg4510 mice it is also interesting to consider neuron-specific genes that are not downregulated in what is a falling total neuronal population; these might represent transcripts that are actually upregulated in response to neuropathology in neuronal cells. Third, compared with the rTg4510 model, relatively few transcriptional changes were observed in J20 mice, potentially reflecting the slower and later accumulation of pathology (Harris et al., 2010b), as well as the potential absence of neurodegeneration, in the entorhinal cortex in this model. This is confirmed by our immunohistochemistry data, which revealed much lower amyloid pathology in the entorhinal cortex than hippocampus tissue from the same J20 individuals (Figures 1E and 1F), and by our targeted qPCR data on selected genes, which highlighted stronger effects in the hippocampus than the entorhinal cortex (Figure S8); future work should focus on more systematic transcriptional profiling in other brain regions more directly affected in the early stages of amyloid pathology. Interestingly, however, we found that effect sizes for the transcripts identified as being progressively dysregulated in rTg4510 and J20 mice were significantly correlated across both models, suggesting common transcriptional mechanisms involved in both tau and amyloid pathology.

In summary, we observe widespread transcriptional changes in the entorhinal cortex paralleling the progression of AD pathology. Our data suggest that the altered expression of multiple genes, including several known AD risk genes, is robustly associated with the accumulation of tau, with tau-associated co-expression networks overlapping those altered in human AD cortex. Our data provide further support for an immune-response component in the accumulation of tau and reveal molecular pathways associated with the progression of AD neuropathology.

## STAR★Methods

### Key Resources Table

**Table:** 

REAGENT or RESOURCE	SOURCE	IDENTIFIER
**Antibodies**		

Mouse monoclonal PG5	P. Davies Albert Einstein College of Medicine	Cat#PG5, RRID: AB_2716729
Mouse monoclonal AT8	Eli Lilly	N/A
Mouse monoclonal biotinylated 3D6	Eli Lilly	N/A
Rabbit polyclonal anti-Iba1	Wako	Cat#019-19741, RRID: AB_839504
Biotinylated goat anti-mouse IgG	Vector labs	Cat# BA-9200, RRID: AB_2336171
Biotinylated goat anti-rabbit IgG	Vector labs	Cat# BA-1000, RRID: AB_2313606

**Biological Samples**		

Mouse brain tissue samples	This paper	N/A

**Critical Commercial Assays**		

TruSeq Stranded mRNA Sample Prep Kit	Illumina	Cat#20020595

**Deposited Data**		

Raw RNA-seq data	This paper	GEO: GSE125957
Results for all expressed transcripts	This paper	www.epigenomicslab.com/ADmice
Mouseac datasets	Matarin et al., 2015	GEO: GSE64398

**Experimental Models: Organisms/Strains**		

Mouse: rTg(tet-o-TauP301L)4510	Envigo	N/A
Mouse: B6.Cg-Zbtb20Tg(PDGFB-APPSwInd)20Lms/2Mmjax	Envigo	JAX: 006293

**Oligonucleotides**		

Abca8a	Thermo Fisher	Assay ID: Mm00462440_m1
Apoe	Thermo Fisher	Assay ID: Mm01307193_g1
Blnk	Thermo Fisher	Assay ID: Mm01197846_m1
Car4	Thermo Fisher	Assay ID: Mm00483021_m1
Ccdc80	Thermo Fisher	Assay ID: Mm00458971_m1
Cd33	Thermo Fisher	Assay ID: Mm00491152_m1
Cd68	Thermo Fisher	Assay ID: Mm03047343_m1
Clec7a	Thermo Fisher	Assay ID: Mm01183349_m1
Clu	Thermo Fisher	Assay ID: Mm01197002_m1
Cst7	Thermo Fisher	Assay ID: Mm00438351_m1
Frmd4a	Thermo Fisher	Assay ID: Mm00552725_m1
Gfap	Thermo Fisher	Assay ID: Mm01253033_m1
Gpr17	Thermo Fisher	Assay ID: Mm02619401_s1
Hspa5	Thermo Fisher	Assay ID: Mm00517691_m1
Htr1a	Thermo Fisher	Assay ID: Mm00434106_s1
Itgax	Thermo Fisher	Assay ID: Mm00498701_m1
Picalm	Thermo Fisher	Assay ID: Mm00525455_m1
Pld3	Thermo Fisher	Assay ID: Mm01171272_m1
Trem2	Thermo Fisher	Assay ID: Mm04209424_g1
Actb	Thermo Fisher	Assay ID: Mm02619580_g1
Eif4a2	Thermo Fisher	Assay ID: Mm01730183_gH
Gapdh	Thermo Fisher	Assay ID: Mm99999915_g1

**Software and Algorithms**		

fastq-mcf	Aronesty, 2011	https://github.com/ExpressionAnalysis/ea-utils/blob/wiki/FastqMcf.md
STAR	Dobin et al., 2013	https://github.com/alexdobin/STAR
featureCounts	Liao et al., 2014	http://subread.sourceforge.net/
DESeq2	Love et al., 2014	http://bioconductor.org/packages/release/bioc/html/DESeq2.html
WGCNA	Langfelder and Horvath, 2008	https://horvath.genetics.ucla.edu/html/CoexpressionNetwork/Rpackages/WGCNA/
GOseq	Young et al., 2010	https://bioconductor.org/packages/release/bioc/html/goseq.html

**Other**		

Human co-expression modules in the dorsolateral prefrontal cortex (DLPFC) and temporal cortex (TCX)	Logsdon et al., 2019	N/A
Supporting code	This paper	https://git.exeter.ac.uk:443/ic322/ad-mice-rna-seq-cell-reports

### Lead Contact and Materials Availability

Further information and requests for resources and reagents should be directed to and will be fulfilled by the Lead Contact, Prof Jonathan Mill (j.mill@exeter.ac.uk). This study did not generate new unique reagents.

### Experimental Model and Subject Details

All animal procedures were carried out at Eli Lilly and Company, in accordance with the UK Animals (Scientific Procedures) Act 1986 and with approval of the local Animal Welfare and Ethical Review Board. Only female mice were used in this study because of their elevated and more progressive pathology compared to males (Blackmore et al., 2017, Yue et al., 2011), and to minimize heterogeneity between samples. rTg4510 (rTg(tet-o-TauP301L)4510) (Ramsden et al., 2005, Santacruz et al., 2005), licensed from the Mayo Clinic (Jacksonville, FL, USA), were bred on a mixed 129S6/SvEvTac + FVB/NCrl background (heterozygous tau responder x heterozygous tTA effector). Bi-transgenic (CC, here referred as TG) female mice and littermate controls (WW, here identified as WT) at ages 2, 4, 6 and 8 months-old (n = 9-10 animals per group) were used for this study. J20 (B6.Cg-Zbtb20Tg(PDGFB-APPSwInd)20Lms/2Mmjax) (Harris et al., 2010a, Mucke et al., 2000), licensed from Gladstone Institute (San Francisco, California, United States), with founder mice purchased from MMRRC at The Jackson Laboratory (Bar Harbor, Maine, United States), were bred on a C57BL/6JOlaHsd background (parental generation: hemizygous male x wild-type female). Hemizygous (here identified as TG) females and littermate controls (WT) at ages 6, 8, 10 and 12 months of age (n = 9-10 animals per group) were used for this study. All mice were bred and delivered to Eli Lilly and Company (Windlesham, UK) by Envigo (Loughborough, UK). At Eli Lilly, animals were housed under standard conditions (constant temperature and humidity) with a 12h light/dark cycle in individually ventilated cages (up to 5 animals per cage), with free access to food (Teklad irradiated global rodent diet (Envigo, United Kingdom)) and water.

### Method Details

#### Brain samples

Mice were terminally anaesthetized with pentobarbital (intraperitoneal injection) and transcardially perfused with phosphate-buffered saline (PBS), and their brains were collected. The entorhinal cortex and hippocampus were dissected from the left brain hemisphere on wet ice (according to Heffner et al., 1980) and snap-frozen on dry ice for subsequent transcriptional analysis. The right brain hemisphere was immersed in 10% buffered formalin for fixation (7-8 days) and processed for subsequent immunohistochemistry pathology assessments.

#### Histopathology

The right hemisphere from all animals was processed using a Tissue TEK® VIP processor (GMI Inc) and embedded in paraffin wax. 6 μm serial sagittal sections (from bregma 0.84 to 3.00) were obtained using rotary microtomes (HM 200 from Ergostar and HM 355S from Thermo Scientific), with sections mounted on glass slides (two sections per slide). Negative and positive controls were used for each immunohistochemistry experiment. Deparaffinisation of the tissue was achieved using xylene (Fisher Scientific), followed by 70% ethanol (industrial methylated spirit, Fisher Scientific) and deionised water for rehydration of the sections. Heat induced epitope retrieval was performed in a PT Module (Thermo Scientific) containing citrate buffer (dilution 1:100). Samples were blocked using normal goat serum (Vector labs, catalog number S-1000). To assess tau pathology, we used mouse monoclonal PG5 (provided by Peter Davies from Albert Einstein College of Medicine, Bronx, NY, USA) (Jicha et al., 1999) as the primary antibody (1:8000), which recognizes tau phosphorylated at Ser409, and biotinylated goat anti-mouse IgG (Vector labs, catalog number BA-9200, lot number 2B0324) as the secondary antibody (1:200), as previously described (Ahmed et al., 2014). Using this same protocol, we also assessed tau pathology with the mouse monoclonal AT8 (provided by Eli Lilly, 1:4000) primary antibody specific for tau phosphorylated at Ser202 and Thr205. To assess amyloid pathology, we used mouse monoclonal biotinylated 3D6 (b3D6, provided by Eli Lilly, 1:1000), which binds to the amino acids 1-5 in amyloid beta (Aβ) (Demattos et al., 2012). To quantify Iba1, we used rabbit polyclonal anti-Iba1 (Wako, catalog number 019-19741, lot number LKG5732) as the primary antibody (diluted 1:6000), and biotinylated goat anti-rabbit IgG (Vector labs, catalog number BA-1000, lot number ZB1007) as the secondary antibody (diluted 1:200). All samples for each mouse model were immunostained together in an autostainer (Autostainer 720 for PG-5 and 720N for b3D6, Thermo Scientific). For detection we undertook enzymatic labeling using peroxidase (Vectastain Elite ABC HRP Reagent, Vector Laboratories) and DAB substrate (Vector Laboratories). Images were digitised with Scanscope AT slide scanner (Aperio) at 20x magnification. Visualization of the digitized tissue sections and delineation of the regions of interest (hippocampus, cortex, thalamus and entorhinal cortex) were achieved using Imagescope software (version 12.2.1.5005; Aperio). Positivity was quantified automatically using a positive pixel algorithm calibrated to ignore non-specific staining, and the burden of tau or amyloid pathology was expressed as percentage area. Statistical analysis (two-way ANOVA) was performed using R (version 3.4.3).

#### RNA isolation and highly-parallel RNA sequencing

Samples were labeled with anonymized ID codes and processed in batches, blinding genotype from the experimenter/analyst for individual samples. Tissue samples from each model were processed separately and individual samples were randomized to ensure that each group was equally represented in each processing batch. Total RNA from the entorhinal cortex and hippocampus was isolated using the AllPrep DNA/RNA Mini Kit (QIAGEN), with minor modifications to the manufacturer’s protocol. Briefly, we added lysis buffer (containing added β-mercaptoethanol) to each tissue sample, disrupted the tissue using a homogenizing pestle, and homogenized the lysate using a pipette. The lysate was centrifuged and the supernatant removed and transferred to an AllPrep DNA spin column. After centrifugation, the flow-through was used for RNA purification by mixing with 70% ethanol, running it through the RNeasy spin column (including DNase treatment) and eluting in RNase-free water. RNA quality and quantity for all samples was checked using RNA ScreenTape (Agilent). The optimal eight entorhinal cortex samples for each group (RIN ≥ 8, Tables S1 and S2) were selected for transcriptional profiling (total n = 128 samples; two models (rTg4510/J20) x two groups (TG/WT) x four time-points x eight individual animals per group). Stranded-specific mRNA sequencing libraries were prepared using the TruSeq Stranded mRNA Sample Prep Kit (Illumina) using the Bravo Automated Liquid Handling Platform (Agilent). cDNA libraries were prepared from ∼450ng of total RNA plus ERCC spike-in synthetic RNA controls (Risso et al., 2014) (Ambion, dilution 1:100). Libraries were individually cleaned up using Ampure XP magnetic beads (Beckman Coulter), their concentrations were determined using D1000 ScreenTape System (Agilent), and samples were pooled together to a 2nM concentration, for subsequent sequencing (three pools of 22 samples for J20 samples and one pool of 64 samples for rTg4510 samples). Pooled libraries were quantified using a Qubit Fluorometer (Thermo Fisher Scientific), Tapestation HS ScreenTape System (Agilent Technologies), and qPCR. Final library pools were distributed across twelve HiSeq2500 (Illumina) lanes (six lanes for each model) and subjected to 125bp paired-end sequencing yielding a mean untrimmed read depth of ∼20 million reads/sample (Tables S1 and S2).

#### Validation of specific gene expression differences using qPCR

Total RNA isolated from the entorhinal cortex and the hippocampus (n = 7-8 animals per group) was used for targeted gene expression analysis. Complementary DNA (cDNA) was reverse transcribed using the EvoScript Universal cDNA Master (Roche) and quantitative RT-PCR was performed in duplicate using the QuantStudio 12 K Flex (Applied Biosystems) using TaqMan low-density arrays (TLDA) and preoptimized assays targeting (i) the top-ranked genotype-associated DEGs in each mouse model (*Car4, Gpr17, Blnk, Hspa5, Ccdc80, Abca8a,* and *Htr1a*), (ii) selected top-ranked interaction (Genotype^∗^Age)-associated DEGs (*Gfap, Cd68, Itgax, Clec7a,* and *Cst7*), (iii) genes previously implicated in AD pathology from genetic studies in humans and found to progressively change in our rTg4510 RNA-seq dataset (*Trem2, Pld3, Frmd4a, Clu, Apoe, Picalm,* and *Cd33*), (iv) three housekeeping genes (*Actb, Eif4a2,* and *Gapdh*), and (v) one internal control (18S ribosomal RNA). The full list of qPCR assays used is described in the **Key Resources Table**.

### Quantification and Statistical Analysis

#### RNA-seq data processing

All sequencing data processing was performed on a Unix-based operating system server at the University of Exeter. Raw files were demultiplexed into *FASTQ* files (Phred (Q) ≥ 35, Tables S1 and S2) and checked for potential contamination. The randomized FASTQ files underwent quality control (QC) assessments using *FastQC* (Andrews, 2010) (version 0.11.4). Trimming (ribosomal sequences removal, quality threshold 20, minimum sequence length 35) was performed with f*astqmcf.* (Aronesty, 2011) (version 1.0) and trimmed samples were aligned to the *mm10* (GRCm38.p4) reference mouse genome using *STAR* (Dobin et al., 2013) (version 2.5.3a), with mapping ≥ 85% (Tables S1 and S2). Gene expression quantification (quantification of fragments or templates, hereby referred as read counts) was achieved using *featureCounts* (Liao et al., 2014) (version 1.5.2). Following confirmation of genotype and QC, 7 samples were excluded from subsequent analysis leaving a final number of 121 high-quality RNA-seq datasets (6-8 animals per group).

#### RNA-seq gene expression analysis

All analyses were performed in R (version 3.4.3) unless otherwise stated. Read counts were analyzed for differential expression using the R package DESeq2 (Love et al., 2014) (version 1.16.1) downloaded from Bioconductor (Matarin et al., 2015). DESeq2 uses the raw read counts, applies an internal normalization method, and does estimation of library size, estimation of dispersion, and negative binomial generalized linear model fitting (Love et al., 2014). Datasets were filtered for non-expressed and lowly expressed genes (minimum of 6 counts across all samples). We were interested in detecting both genotype effects and progressive changes across age between the transgenic and wild-type samples. We used the following statistical model, including main effects for both Genotype and Age (both coded as categorical variables) and an interaction between these two terms:

Geneexpression=Genotype+Age+Genotype∗Age

To identify significant genotype effects a Wald test was used, and to identify significant effects of age and interaction effects (i.e., Genotype^∗^Age) we used the likelihood-ratio test, both applied with the *DESeq* function from the *DESeq2* package (Love et al., 2014). To test for associations between gene expression and measures of neuropathology quantified using immunohistochemistry, we fitted linear models using *DESeq* and used a Wald test to calculate *P value*s. These models were fitted separately for neuropathology data measured in the entorhinal cortex and hippocampus for each individual. *P value*s were adjusted for multiple testing using the false discovery rate (FDR) method (also known as Benjamini and Hochberg correction) (Benjamini and Hochberg, 1995) implemented with the R function *p.adjust*; FDR values < 0.05 were defined as significant. Potential differences in proportions of upregulated versus downregulated genes as well as overlapping fold changes in both models were interrogated using the binomial test. Functional annotation and gene ontology analyses were done with GOseq (Young et al., 2010) (1.30.0), based on genes with FDR < 0.05.

#### Quantifying human transgene expression

Mouse and human *App*/*APP* and *Mapt*/*MAPT* sequences were compared using BLAT (Kent, 2002) for divergent transcript sequences representing specific mouse and human gene sequences. Two 200bp regions spanning 4 exons were chosen as representative mouse-specific *App*. Similar regions consisting of two 200bp exonic regions were also chosen for human *APP*. Mouse-specific *Mapt* and human-specific *MAPT* sequences were chosen from a 2kb region present in the 3′UTR. Using *Bowtie2* (Langmead et al., 2009) (version 2.3.4.3), indices based on these sequences were then built, and alignments were performed using the *FASTQ* read 1 sequences. Counts of read alignments for mouse and human specific indices were then plotted as a ratio of unique (mouse or human) reads relative to the total number of input reads.

#### Comparison with RNA-seq data from the Mouseac database

RNA-seq data (transcripts per million, TPM) from two mouse models (Matarin et al., 2015, Salih et al., 2019) (TAU (CaMKII-MAPTP301L) and TAS10 (SwAPP, K670N/M671L)) were downloaded from the Mouseac online database (www.mouseac.org), with corresponding detailed phenotypic data downloaded from GEO (Barrett et al., 2013, Edgar et al., 2002) (accession number GSE64398). Only genes identified as differentially expressed (FDR < 0.05) in our analysis (for rTg4510 and J20 mice) were selected for further statistical analysis. The TPM value for each gene was log transformed (log2(x+1)) and the same linear regression model described above (Geneexpression=Genotype+Age+Genotype∗Age), using ANOVA to test for significant differences associated with either the Genotype, Age, or Genotype^∗^Age terms, was used. *P value*s were corrected for the number of genes compared across datasets using Bonferroni correction. Potential differences in proportions of upregulated versus downregulated genes as well as overlapping fold changes in both models were interrogated using the binomial test.

#### Co-expression network analysis

Using weighted co-expression network analysis (Langfelder and Horvath, 2008, Zhang and Horvath, 2005) (WGCNA) (version 1.63), we constructed a signed co-expression network for each mouse model using log transformed counts from all samples. Logarithmic transformation of raw counts was achieved using the *rlog* function from DESeq2, which minimizes variability in genes with low counts (Love et al., 2014). We checked the data for missing values and outliers, and removed one sample from the analysis for the rTg4510 dataset (flagged as an outlier) before building the networks. Signed WGCNA co-expression networks were built using the lowest power for which the scale-free topology fit index curve flattened out after reaching 0.90 resulting in a soft-threshold power of 10 and 9 for rTg4510 and J20 datasets, respectively, and a minimum module size of 30. For each module of highly interconnected genes, color-labeled according to the WGCNA conventions, we calculated the module eigengenes (MEs) as the first principal component of the expression matrix, which provide a representative expression profile for each module (Langfelder and Horvath, 2008). In order to identify modules significantly associated with pathology burden, we calculated correlation coefficients between these MEs with the available pathology data and explored the most significant associations. In addition we used the same linear regression model as described for the gene-level analysis (ME=Genotype+Age+Genotype∗Age) using ANOVA to test for significant differences due to either the Genotype, Age, or Genotype^∗^Age terms. *P value*s were corrected for multiple comparisons using the Bonferroni correction, to correct for 18 modules in the rTg4510 (statistical threshold was adjusted to 0.05/18 = 0.0028) and 21 modules in the J20 (statistical threshold was adjusted to 0.05/21 = 0.0024) mice. We used the *GOseq* R package (version 1.30.0) to perform functional annotation and gene ontology (GO) analyses for each module, where significant pathways were selected using an FDR threshold of 0.05 as previously described (Young et al., 2010). We used *Cytoscape* (Shannon et al., 2003) (version 3.7.0) for network visualization using the topological overlap matrix for the log transformed expression data.

#### Comparison with human co-expression networks

The six rTg4510-associated co-expression modules identified in this study (“salmon,” “turquoise,” “purple,” “yellow,” “light-cyan,” and “red”), and AD-associated human co-expression modules in the dorsolateral prefrontal cortex (DLPFC) and temporal cortex (TCX) (Logsdon et al., 2019), were reduced to contain only mouse-human homologs as defined by Ensembl (Zerbino et al., 2018) (accessed on 14/11/2018). The level of overlap between gene members of each pair of modules was assessed via a hypergeometric test using the R (version 3.5.1) function *phyper*. *P value*s were corrected for multiple comparisons using Bonferroni correction in a tissue-specific manner, where only the set of raw *P value*s related to DLPFC (statistical threshold was adjusted to 0.05/24 = 0.0021) or TCX (statistical threshold was adjusted to 0.05/30 = 0.0017) modules’ overlap were considered. Using *GOseq* (version 1.30.0), we performed functional annotation and GO analyses for the common genes in each overlapping pair of modules.

#### Targeted qPCR analysis

The abundance of each test gene was determined using the comparative Ct method (Pfaffl, 2001), expressed relative to the mean of the three housekeeping genes selected using *RefFinder* software (Xie et al., 2012) that determines the best stably expressed reference gene(s). A linear regression model using ANOVA to test for significant differences associated with either Genotype, Age, or Genotype^∗^Age terms was used (ΔCt=Genotype+Age+Genotype∗Age), with *P value*s adjusted for multiple testing using the FDR method implemented with the R function *p.adjust*.

### Data and Code Availability

Raw RNA-seq data has been deposited in GEO under accession number GSE125957. Results for all expressed transcripts in both transgenic models are available to download from www.epigenomicslab.com/ADmice. The code supporting the current study is available at https://git.exeter.ac.uk:443/ic322/ad-mice-rna-seq-cell-reports.

## Supplemental Information

Document S1. Figures S1–S10

Table S1. Detailed Information and RNA-Seq Quality Metrics for the rTg4510 and J20 Samples Included in This Study, Related to STAR Methods

Table S2. Differentially Expressed Genes Associated with rTg4510 and with J20 Genotype (FDR < 0.05), Related to Figure 2 Results for all transcripts are available to download at www.epigenomicslab.com/ADmice.

Table S3. Results from Targeted qPCR Assays for Genotype-Associated Expression Differences and for Genes Differentially Expressed with Age in TG Mice Identified Using RNA-Seq, related to Figures 2, 3, 4, and 5 Each transcript was quantified in the entorhinal cortex and hippocampus.

Table S4. Differentially Expressed Genes across Age Groups in rTg4510 and J20 TG Mice (FDR < 0.05), Related to Figures 3, 4, and 5 Results for all transcripts are available to download at www.epigenomicslab.com/ADmice. Rows highlighted in green denote genes for which an age effect irrespective of genotype is observed. All other genes show are characterized by age changes exacerbated by genotype.

Table S5. Differentially Expressed Genes Associated with Tau or Amyloid Pathology Measured by Immunohistochemistry in Entorhinal Cortex and Hippocampus Samples from rTg5410 and J20 Mice (FDR < 0.05), Related to Figure 4 Results for all transcripts are available to download at www.epigenomicslab.com/ADmice.

Table S6. Gene Ontology Categories Enriched among Genes Characterized by Progressively Altered Gene Expression, and within the Different WGCNA Modules, in rTg4510 Mice, Related to Figures 3, 4, 5, 6, and 7 Gene ontology results for genes within the rTg4510 turquoise module overlapping with those in the “DLPFC-blue” and “TCX-turquoise” human modules are also shown.

Table S7. Statistics for rTg4510 Genotype-Associated Genes in the TAU Model from the Mouseac Dataset, and for Genes Progressively Altered in rTg4510, in the TAU and TAS10 Models from the Mouseac Dataset, Related to Figures 2, 3, 4, and 5

Table S8. Co-expression Modules Significantly Associated with rTg4510 Genotype and Tau Progression, Related to Figures 6 and 7

Table S9. Intra-modular Connectivity for Genes in the Turquoise, Yellow, and Red rTg4510 WGCNA Modules, Related to Figures 6 and 7

Table S10. Overlaps between rTg4510 Co-expression Modules and AD-Associated Modules in the Human Dorsolateral Prefrontal Cortex (DLPFC) and in the Human Temporal Cortex (TCX), Related to Figures 6 and 7

Document S2. Article plus Supplemental Information
