# Supplementary material for: Transcriptional Signatures of Tau and Amyloid Neuropathology
Source: Cell Rep. 2020 Feb 11;30(6):2040–2054.e5. doi: 10.1016/j.celrep.2020.01.063 (PMC7016505; doi:10.1016/j.celrep.2020.01.063)
Supplement: Document S1. Figures S1–S10 [file mmc1.pdf]

Cell Reports, Volume *30*

Supplemental Information

Transcriptional Signatures of Tau

and Amyloid Neuropathology

Isabel Castanho, Tracey K. Murray, Eilis Hannon, Aaron Jeffries, Emma Walker, Emma Laing, Hedley Baulf, Joshua Harvey, Lauren Bradshaw, Andrew Randall, Karen Moore, Paul O'Neill, Katie Lunnon, David A. Collier, Zeshan Ahmed, Michael J. O'Neill, and Jonathan Mill

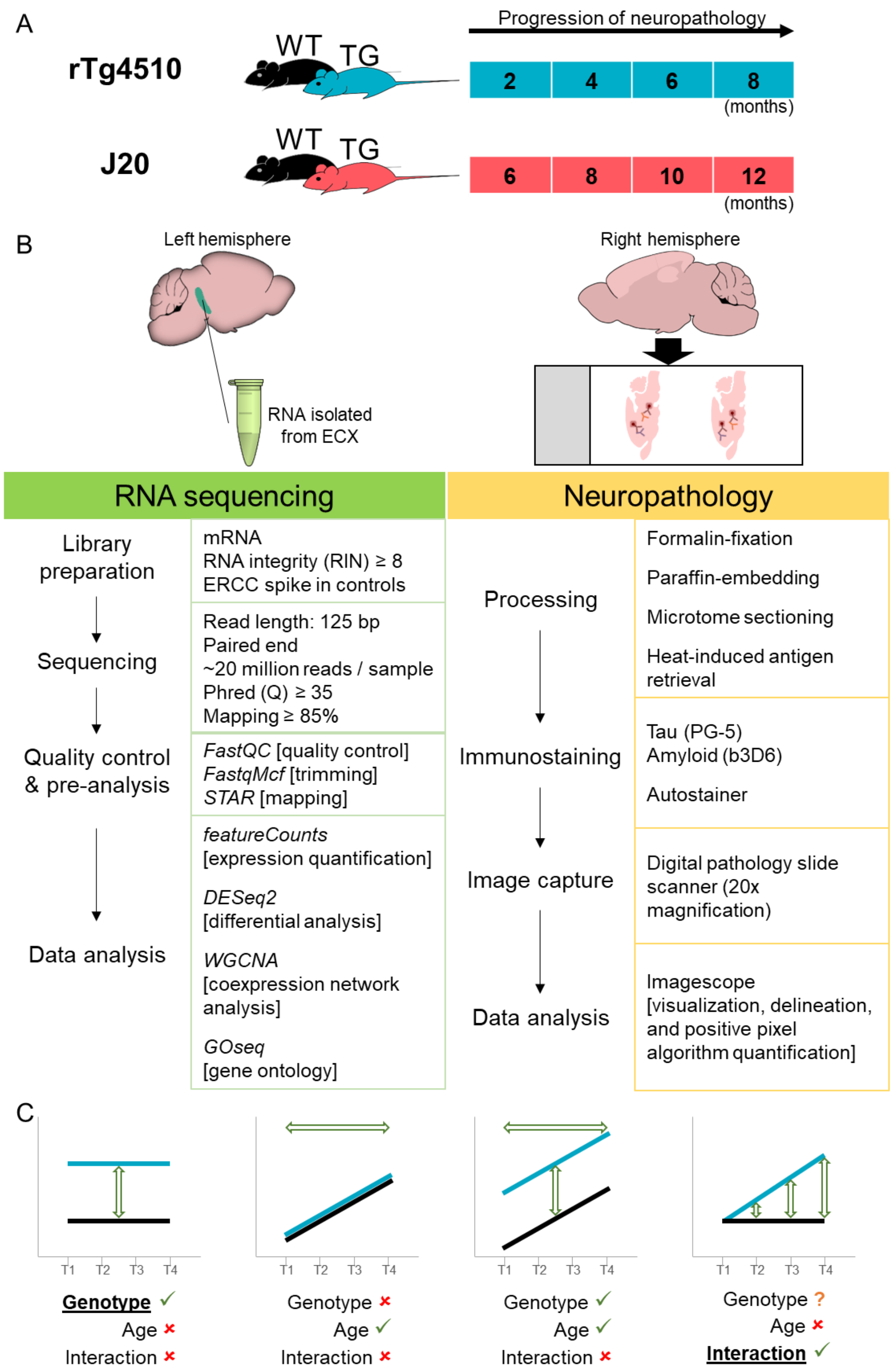

Figure S1. An overview of the experimental approach used in this study, related to STAR Methods. (A) To investigate transcriptional signatures of tau pathology we used the rTg4510 transgenic mouse line, which overexpresses a human mutant (P301L) form of the microtubule-associated protein tau (MAPT). To investigate amyloid pathology, we used the J20 transgenic mouse line, which expresses a mutant (K670N/M671L and V717F) form of the human amyloid precursor protein (APP). Tissue was collected from transgenic (TG) and wild type (WT) control mice at four time points. **(B)** From each mouse, the entorhinal cortex (ECX) was dissected from the left hemisphere and used for RNA isolation and subsequent RNA-seq analysis. The right hemisphere was fixed and used to quantify the progression of neuropathology across multiple brain regions using immunohistochemistry. **(C)** Our analyses focused primarily on i) identifying transcriptional differences associated with genotype and ii) identifying temporal changes in gene expression in TG mice paralleling the development of neuropathology (i.e. interactions between genotype and age).

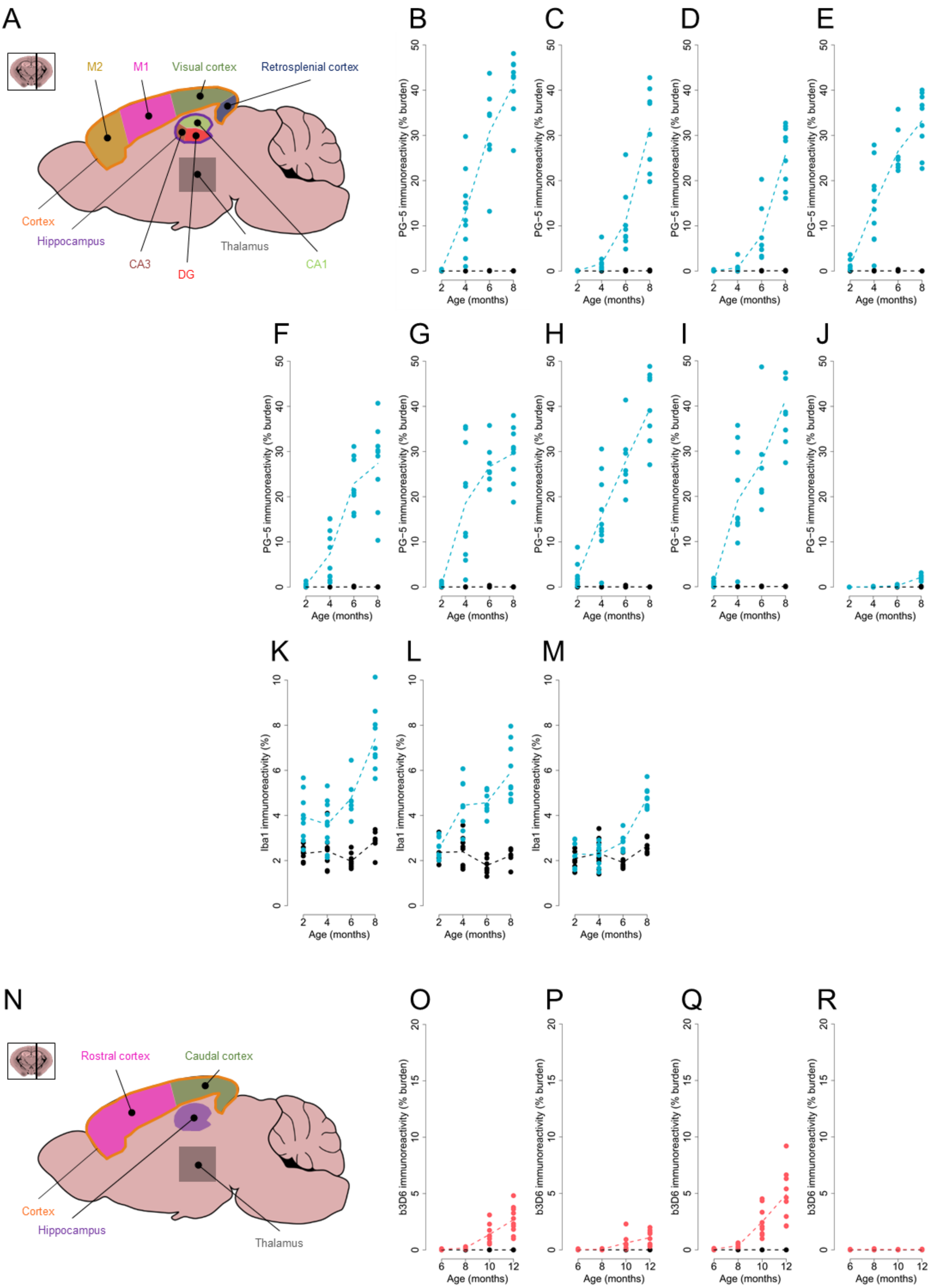

Figure S2. Progressive tau neuropathology in rTg4510 mice, and progressive amyloid pathology in J20 mice, related to Figure 1. (A) The anatomical regions of the brain tested for tau pathology: whole hippocampus (purple line), CA1 subregion of the hippocampus (light green area within the purple line), CA3 subregion of the hippocampus (brown area within the purple line), dentate gyrus (DG) subregion of the hippocampus (light red area within the purple line), whole cortex (orange line), secondary motor cortex (M2, dark yellow area within the orange line), primary motor cortex (M1, magenta area within the orange line), visual cortex (dark green area within the orange line), retrosplenial cortex (dark blue are within the orange line), thalamus (dark

grey square). **(B-J)** Quantification of PG-5 immunoreactivity in each tested region: **(B)** CA1 (total n = 74 animals, 9-10 animals per group, factorial ANOVA, F(3,67) = 68.86, *P* = 1.96E-20), **(C)** CA3 (total n = 73 animals, 8-10 animals per group, factorial ANOVA, F(3,66) = 64.22, *P* = 1.51E-19), **(D)** DG (total n = 73 animals, 8-10 animals per group, factorial ANOVA, F(3,66) = 67.42, *P* = 4.58E-20), **(E)** whole cortex (total n = 72 animals, 7-10 animals per group, factorial ANOVA, F(3,65) = 53.33, *P* = 1.65E-17), **(F)** M2 (total n = 74 animals, 8-10 animals per group, factorial ANOVA, F(3,67) = 44.90, *P* = 5.01E-16), **(G)** M1 (total n = 73 animals, 8-10 animals per group, factorial ANOVA, F(3,66) = 29.28, *P* = 3.74E-12), **(H)** visual cortex (total n = 73 animals, 8-10 animals per group, factorial ANOVA, F(3,66) = 55.22, *P* = 5.64E-18), **(I)** retrosplenial cortex (total n = 73 animals, 8-10 animals per group, factorial ANOVA, F(3,66) = 38.38, P = 1.79E-14). **(J)** As expected, the thalamus, which is relatively protected from aggressive tau pathology, was characterized by relatively low levels of tau. Despite this, TG mice demonstrated significant differences in late stages (total n = 74 animals, 8-10 animals per group, factorial ANOVA, F(3,67) = 111.48, *P* = 5.41E-26). rTg4510 transgenic (TG, blue) female mice compared to wild type (WT, black) littermate control mice. **(K-M)** Quantification of Iba1 immunoreactivity in each tested region: **(K)** hippocampus (total n = 70 animals, 7-10 animals per group, factorial ANOVA, F(3,62) = 12.60, P = 1.56E-06), **(L)** cortex (total n = 70 animals, 7-10 animals per group, factorial ANOVA, F(3,62) = 18.13, P = 1.47E-08), **(M)** thalamus (total n = 70 animals, 7-10 animals per group, factorial ANOVA, F(3,62) = 18.85, P = 8.37E-09). rTg4510 transgenic (TG, blue) female mice compared to wild type (WT, black) littermate control mice. **(N)** Brain regions tested: whole hippocampus (purple area), whole cortex (orange line), rostral cortex (magenta area within the orange line), caudal cortex (green area within the orange line), thalamus (dark grey square). **(O-R)** Quantification of b3D6 immunoreactivity in each tested region: **(O)** whole cortex (total n = 73 animals, 8-10 animals per group, factorial ANOVA, F(3,66) = 24.75, *P* = 7.63E-11), **(P)** rostral cortex (total n = 73 animals, 8-10 animals per group, factorial ANOVA, F(3,66) = 10.63, *P* = 8.65E-06), **(Q)** caudal cortex (total n = 74 animals, 8-10 animals per group, factorial ANOVA, F(3,67) = 28.62, *P* = 5.02E-12), **(R)** thalamus (total n = 73 animals, 8-10 animals per group, factorial ANOVA, F(3,66) = 1.58, *P* = 0.20). J20 transgenic (TG, red) female mice compared to wild type (WT, black) littermate control mice.

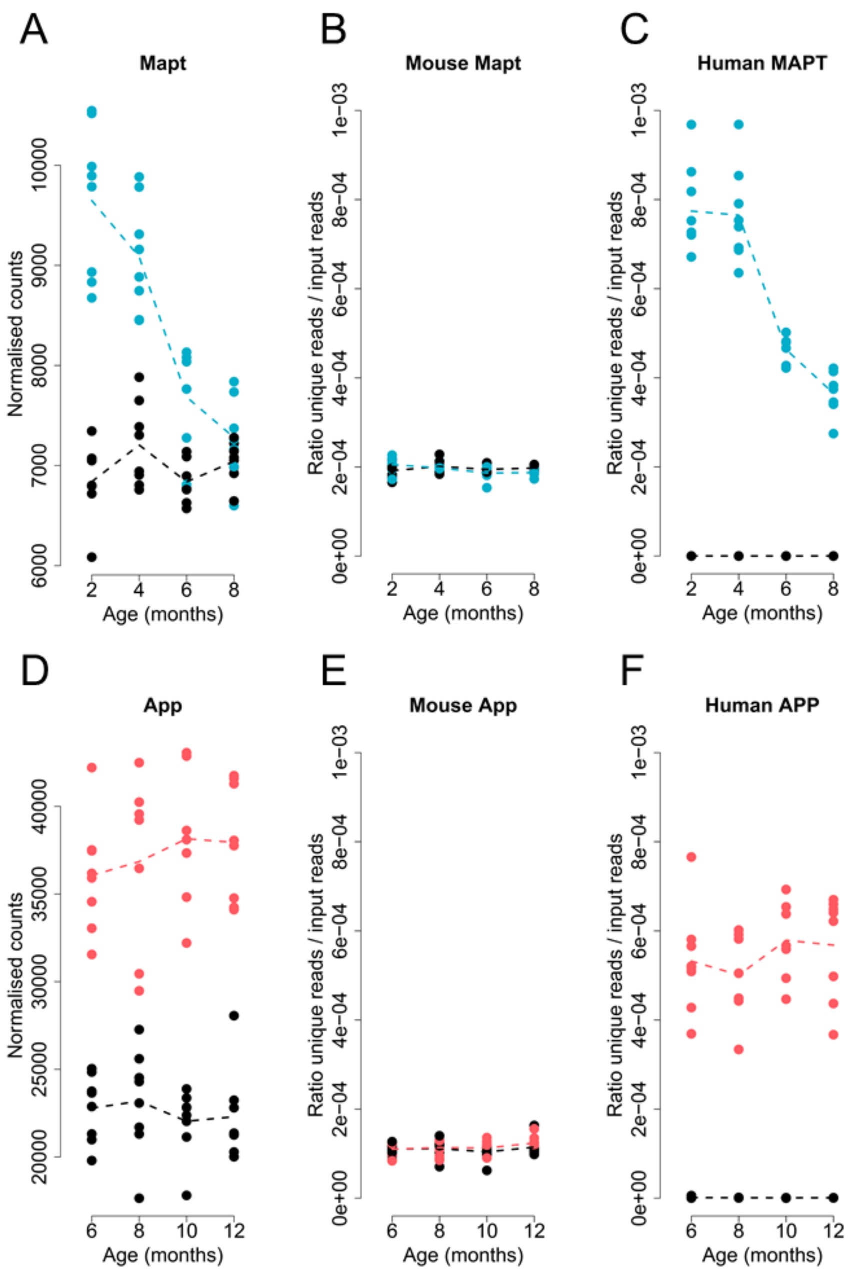

Figure S3. Apparent up-regulation of *Mapt* in rTg4510 mice and *App* in J20 mice results from human-specific sequences only present in transgenic mice, related to Figure 2. (A-C) Apparent up-regulation of *Mapt* in rTg4510 transgenic (TG, blue) female mice compared to wild type (WT, black) littermate control mice (n = 59 animals). **(A)** *Mapt* gene expression from *DESeq2* highlights elevated expression of *Mapt* in TG mice (Wald test, Wald statistic = 11.11, log2 fold change = 0.50, FDR = 7.08E-25). **(B)** Ratio of unique reads that mapped to mouse-specific sections of *Mapt* or **(C)** human-specific sections of *MAPT*. **(D-F)** Apparent up-regulation of *App* in J20 transgenic (TG, red) female mice compared to wild type (WT, black) littermate control mice (n = 62 animals). **(D)** *App* gene expression from *DESeq2* highlights elevated expression of *App* in TG mice (Wald test, Wald statistic = 8.55, log2 fold change = 0.66, FDR = 2.37E-13). **(E)** Ratio of unique reads that mapped to mouse-specific sections of *App* or **(F)** human-specific sections of *APP*. Normalised counts were obtained using *DESeq2*. Dashed lines represent mean paths across age groups.

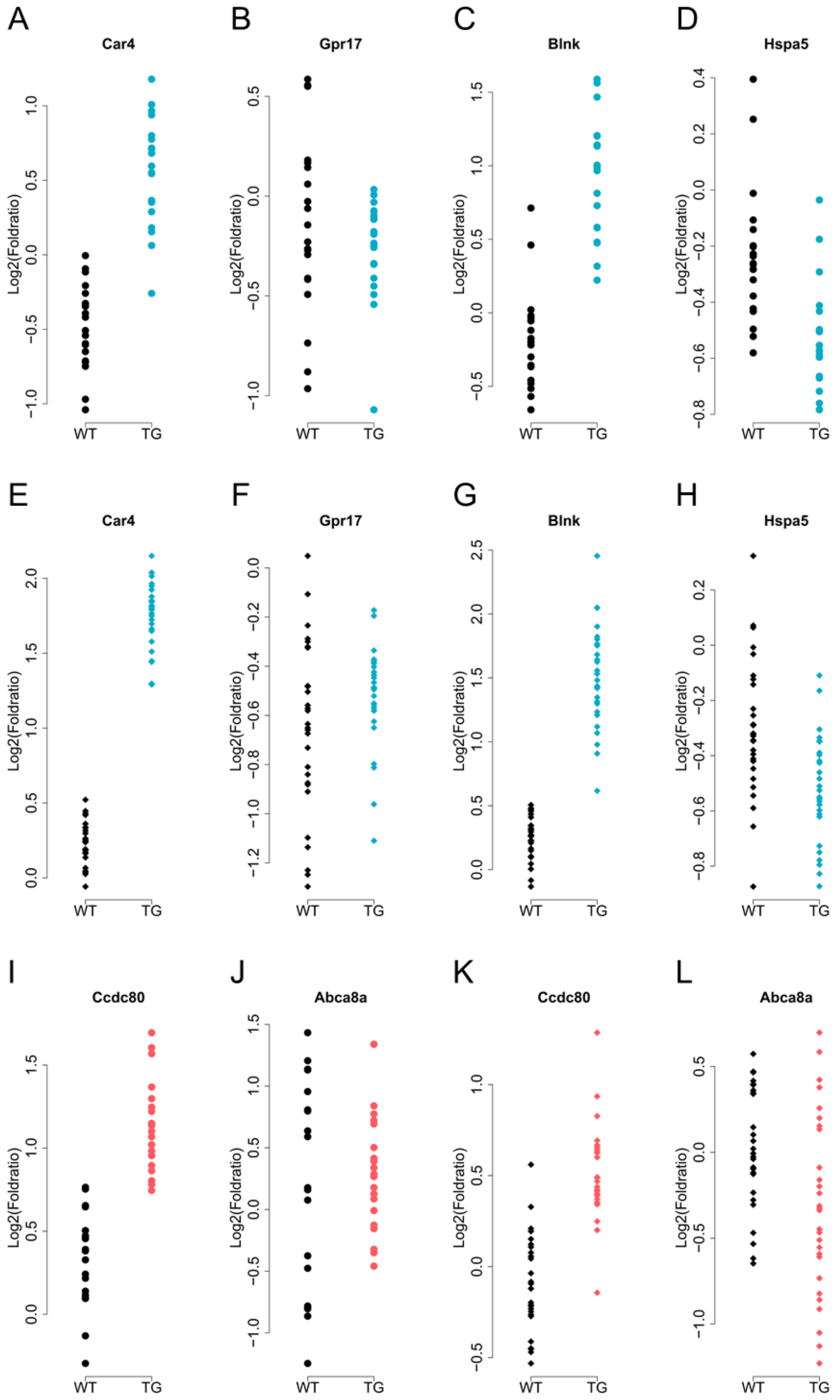

Figure S4. Top-ranked differentially expressed transcripts associated with rTg4510 genotype and with J20 genotype altered in the entorhinal cortex and hippocampus, related to Figure 2. Shown are the results for qPCR validation for rTg4510 mice in entorhinal cortex (**A-D**) and hippocampus (**E-H**), and for J20 mice in entorhinal cortex (**I-J**) and hippocampus (**K-L**). Statistical results are listed in **Supplementary Table 3**. All qPCR reactions were run in duplicate and the abundance of each test gene was determined using the comparative Ct method expressed relative to the mean of the three housekeeping genes (*Actb, Eif4a2,* and *Gapdh*).

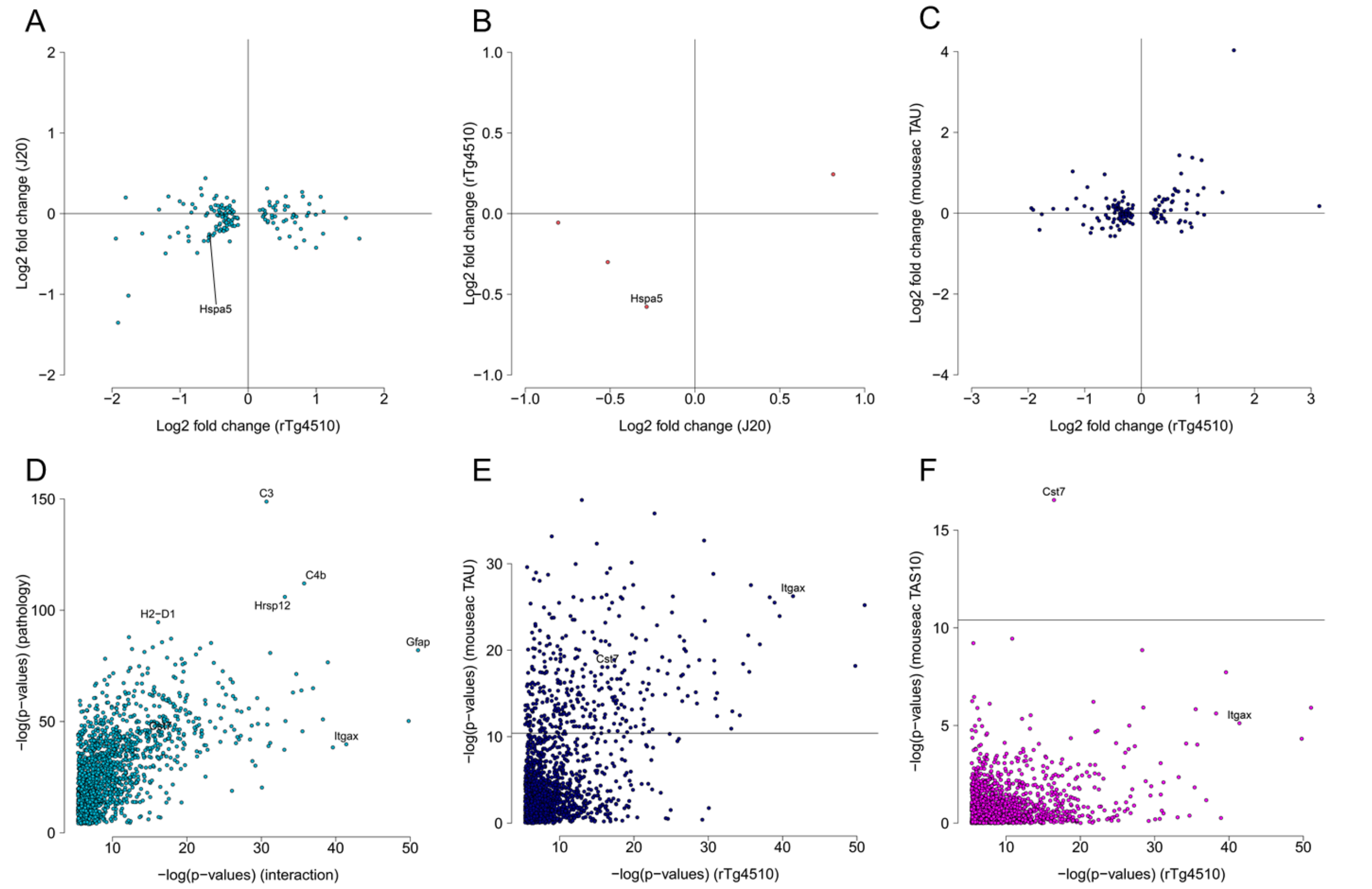

Figure S5. Correlations between the different mouse models, related to Figures 2-5. There is no overall consistency between the rTg4510 and J20 models in effect sizes for genotype-associated differentially-expressed transcripts identified in each individual model: **(A)** Negligible correlation for effect size (Log2 fold change) across models for differentially expressed transcripts (FDR < 0.05) associated with rTg4510 genotype (Pearson correlation, r = 0.18, P = 0.029; exact binomial test, n = 147 transcripts, P = 0.69). **(B)** No correlation for effect size (Log2 fold change) across models for differentially expressed transcripts (FDR < 0.05) associated with J20 genotype (Pearson correlation, r = 0.66, P = 0.23; exact binomial test, n = 4 transcripts, P = 0.13). Despite the overall lack of consistency between rTg4510 and J20, one transcript (*Hspa5*) is differentially downregulated (FDR < 0.05) in the same direction in both models (Tg4510: Wald statistic = -6.16, log2 fold change = -0.58, FDR = 1.37E-06; J20: Wald statistic = -4.36, log2 fold change = -0.28, FDR = 0.049). **(C)** Correlation of effect size (log2 fold change) between rTg4510 genotype-associated genes and the same genes in the TAU mouse model from Mouseac (Pearson correlation, r = 0.33, P = 7.73E-5, exact binomial test, n = 138 transcripts, P = 0.11). **(D)** Association statistics for transcripts differentially expressed with age in rTg4510 TG mice were highly correlated with those from analyses of tau pathology in the hippocampus (Pearson correlation, r = 0.58, P < 1.8E-149). **(E)** Correlation of *P* values between the rTg4510 and TAU mouse models (using transcriptional data from Mouseac) for an analysis of interactions between genotype and age for genes identified as significant in rTg4510 mice (Pearson correlation, r = 0.46, *P* = 1.22E-86, exact binomial test, n = 1640 transcripts, *P* < 2.2e-16). **(F)** Correlation of *P* values between the rTg4510 and TAS10 mouse models (using transcriptional data from Mouseac) for an analysis of interactions between genotype and age for genes identified as significant in rTg4510 mice (Pearson correlation, r = 0.23, *P* = 3.89E-21, exact binomial test, n = 1640 transcripts, *P* < 2.2e-16).

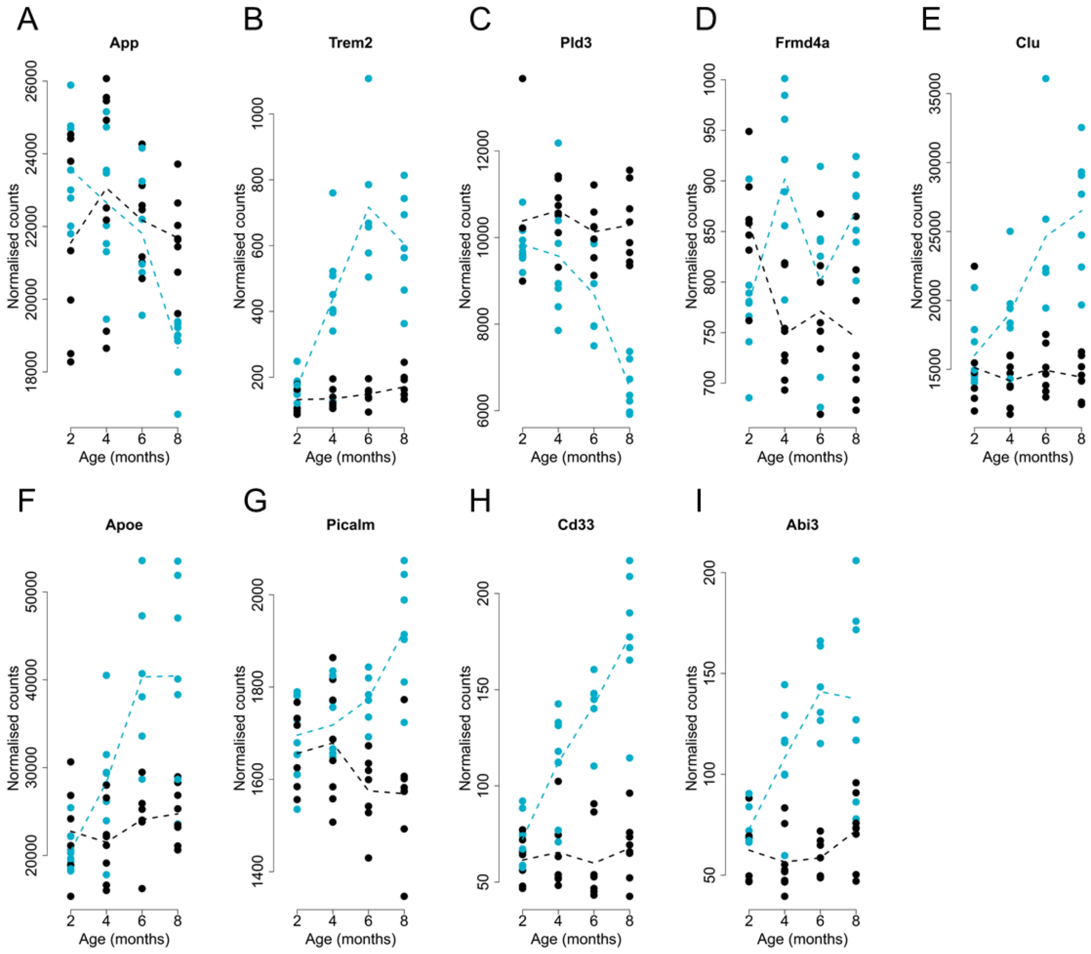

Figure S6. Transcripts progressively altered in rTg4510 mice include many genes implicated in familial and sporadic AD from genetic association studies, related to Figure 3. Genes showing temporal shifts in expression in rTg4510 mice included: **(A)** *App* (Likelihood-ratio test, LRT statistic = 13.88, log2 fold change (2-8 months) = -0.35, FDR = 0.037); **(B)** *Trem2* (Likelihood-ratio test, LRT statistic = 43.82, log2 fold change (2-8 months) = 1.46, FDR = 3.73E-07); **(C)** *Pld3* (Likelihood-ratio test, LRT statistic = 36.80, log2 fold change (2-8 months) = -0.58, FDR = 5.80E-06); **(D)** *Frmd4a* (Likelihood-ratio test, LRT statistic = 27.81, log2 fold change (2-8 months) = 0.36, FDR = 0.00022); **(E)** *Clu* (Likelihood-ratio test, LRT statistic = 27.73, log2 fold change (2-8 months) = 0.80, FDR = 0.00023); **(F)** *Apoe* (Likelihood-ratio test, LRT statistic = 22.99, log2 fold change (2-8 months) = 0.86, FDR = 0.0014); **(G)** *Picalm* (Likelihood-ratio test, LRT statistic = 21.37, log2 fold change (2-8 months) = 0.26, FDR = 0.0025); **(H)** *Cd33* (Likelihood-ratio test, LRT statistic = 27.32, log2 fold change (2-8 months) = 1.14, FDR = 0.00026); and **(I)** *Abi3* (Likelihood-ratio test, LRT statistic = 17.10, log2 fold change (2-8 months) = 0.70, FDR = 0.012). Total n = 59 animals (29 TG, 30 WT). Normalised counts were obtained using *DESeq2*. Dashed lines represent mean paths across age groups. rTg4510 transgenic (TG, blue) mice compared to wild type (WT, black) littermate controls.

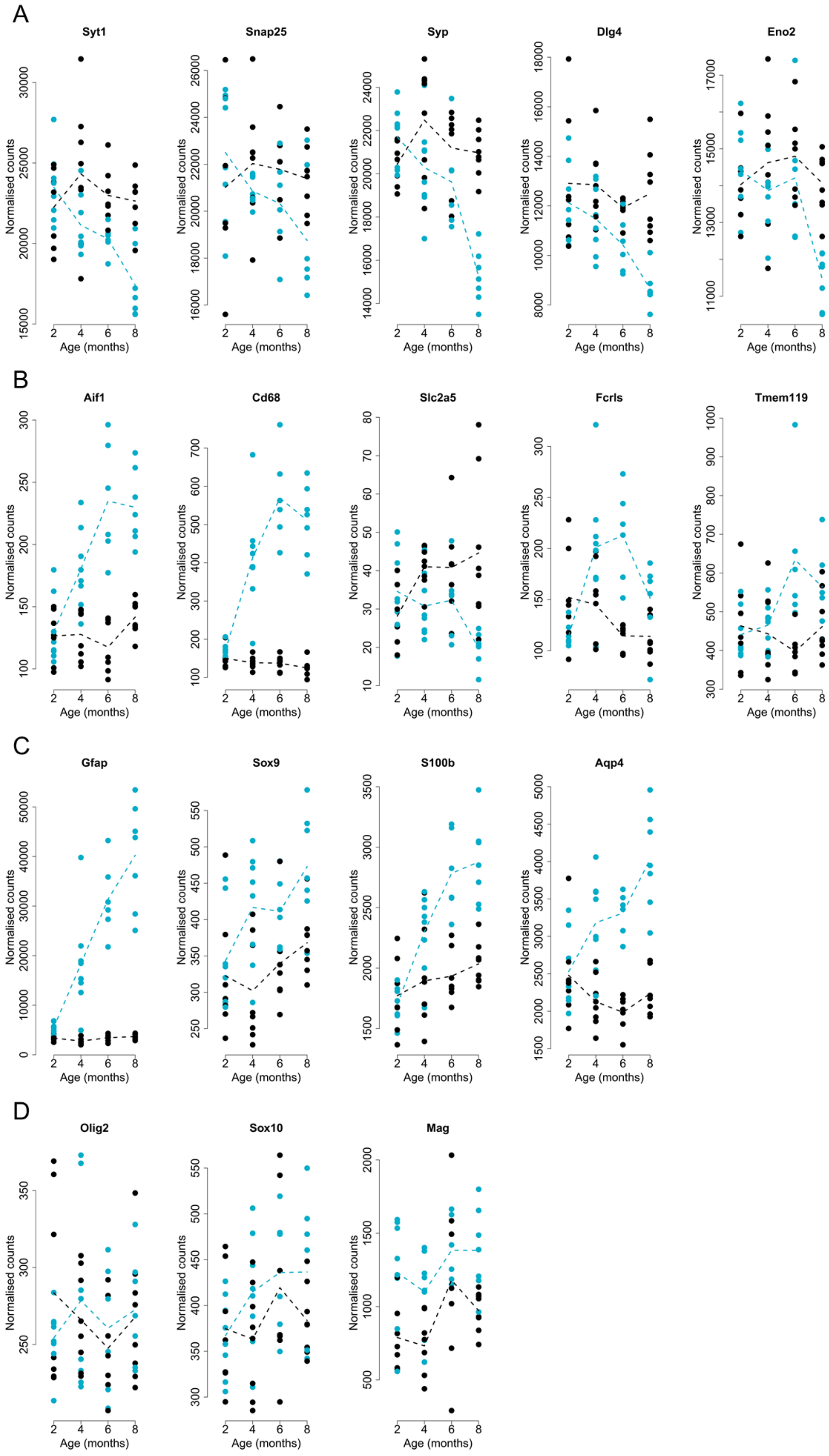

Figure S7. The progression of tau pathology in rTg4510 TG mice is associated with the downregulation of key neuronal markers, with an up-regulation of transcripts associated with both astrocytes and microglia, related to Figure 3. Shown are temporal RNA-seq data for key transcriptional markers of **(A)** neurons (*Sty1*, *Snap25*, *Syp*, *Dig4*, and *Eno2*), **(B)**

microglia/monocytes (*Aif1, Cd68, Slc2a5, Fcrls,* **(C)** astrocytes (*Gfap, Sox9, S100b*, and *Aqp4*), and *Tmem119*), and **(D)** oligodendrocytes (*Olig2, Sox10*, and *Mag*). Normalised counts were obtained using *DESeq2*. Transcript level results are listed in **Supplementary Table 4**. Dashed lines represent mean paths across age groups. rTg4510 transgenic (TG, blue) mice compared to wild type (WT, black) littermate controls.

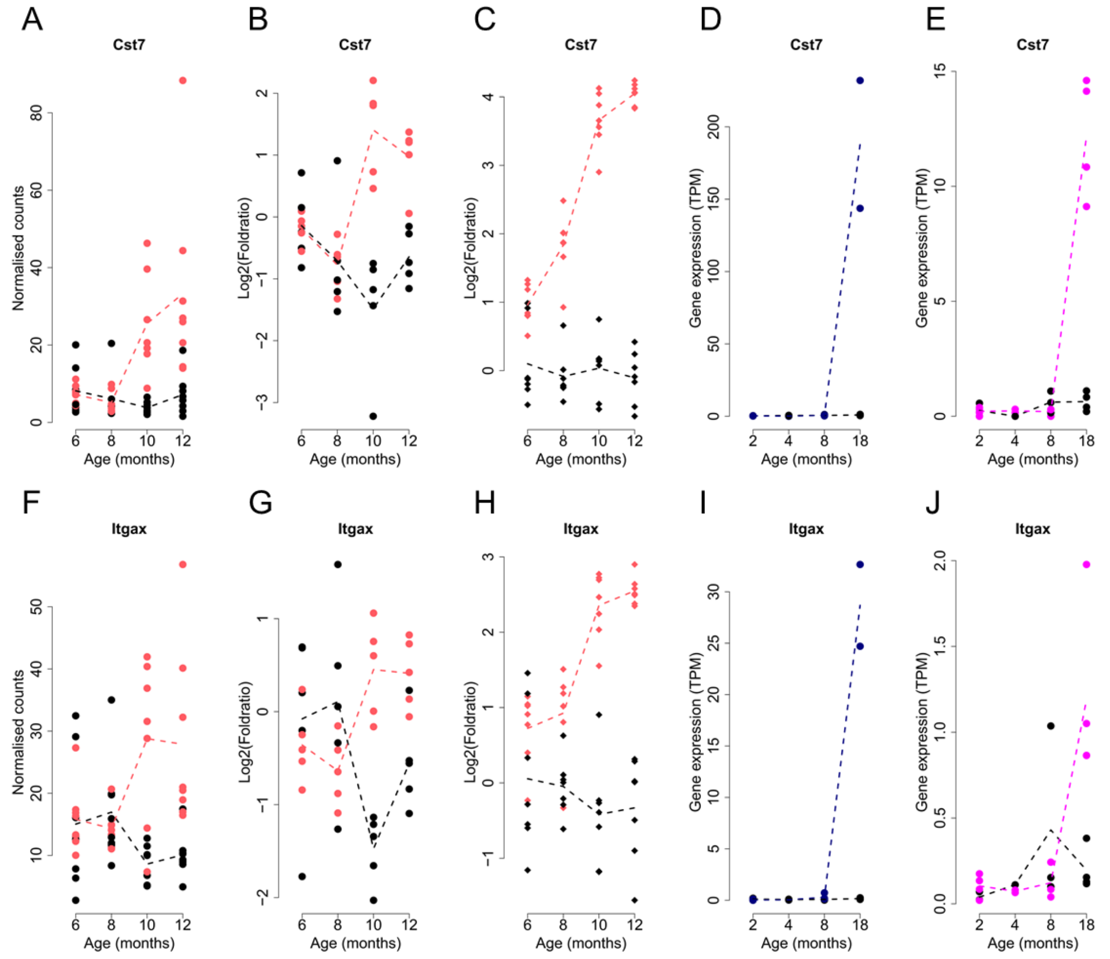

Figure S8. *Cst7* and *Itgax* are similarly altered in all models tested, but are more aggressively upregulated in the hippocampus than the entorhinal cortex in J20 mice, related to Figures 3-5. (A) *Cst7* gene expression in J20 mice (Total n = 62 animals, 6-8 animals per group, Likelihood-ratio test, LRT statistic = 37.37, log2 fold change (6-12 months) = 2.42, FDR = 0.00072). J20 transgenic (TG, red) female mice compared to wild type (WT, black) littermate control mice. **(B)** qPCR validation of progressive differential expression of *Cst7* (Interaction F-statsitc = 11.59, FDR = 0.00061)) in the entorhinal cortex. **(C)** qPCR results for *Cst7* (Interaction F-statistic = 48.16, FDR = 3.76E-13) in the hippocampus. **(D)** *Cst7* in TAU mice from Mouseac (Total n = 25 animals, 2-4 animals per group, linear regression, F(3,16) = 48.73, β = 187.00, *P* = 1.46E-8). TAU transgenic mice (dark blue) compared to wild type (black) controls. **(E)** *Cst7* in TAS10 mice from Mouseac (Total n = 24 animals, 1-4 animals per group, linear regression, F(3,15) = 43.42, β = 11.58, *P* = 6.50E-8). TAS10 transgenic mice (pink) compared to wild type (black) controls. **(F)** The expression of *Itgax* in J20 mice (Total n = 62 animals, 6-8 animals per group, Likelihood-ratio test, LRT statistic = 37.37, log2 fold change (6-12 months) = 2.42, FDR = 0.00072) reflects the changes seen in rTg4510 mice (see **Figure 3C**). J20 transgenic (TG, red) female mice compared to wild type (WT, black) littermate control mice. **(G)** qPCR validation of progressive differential expression of *Itgax* (Interaction F-statistic = 9.12, FDR = 0.0019) in the entorhinal cortex. **(H)** qPCR results for *Itgax* (Interaction F-statistic = 13.09, FDR = 1.31E-5) in the hippocampus. **(I)** *Itgax* in TAU mice from Mouseac (Total n = 25 animals, 2-4 animals per group, linear regression, F(3,16) = 137.86, β = 28.59, *P* = 3.95E-12). TAU transgenic mice (dark blue) compared to wild type (black) controls. **(J)** *Itgax* in TAS10 mice from Mouseac (Total n = 24 animals, 1-4 animals per group, linear regression, F(3,15) = 6.03, β = 0.93, *P* = 0.0060). TAS10 transgenic mice (pink) compared to wild type (black) controls. Dashed lines represent mean paths for each time point. Normalised counts were obtained using *DESeq2*. All qPCR reactions were run in duplicate and the abundance of each test gene was determined using the comparative Ct method expressed relative to the mean of the three housekeeping genes (*Actb, Eif4a2,* and *Gapdh*).

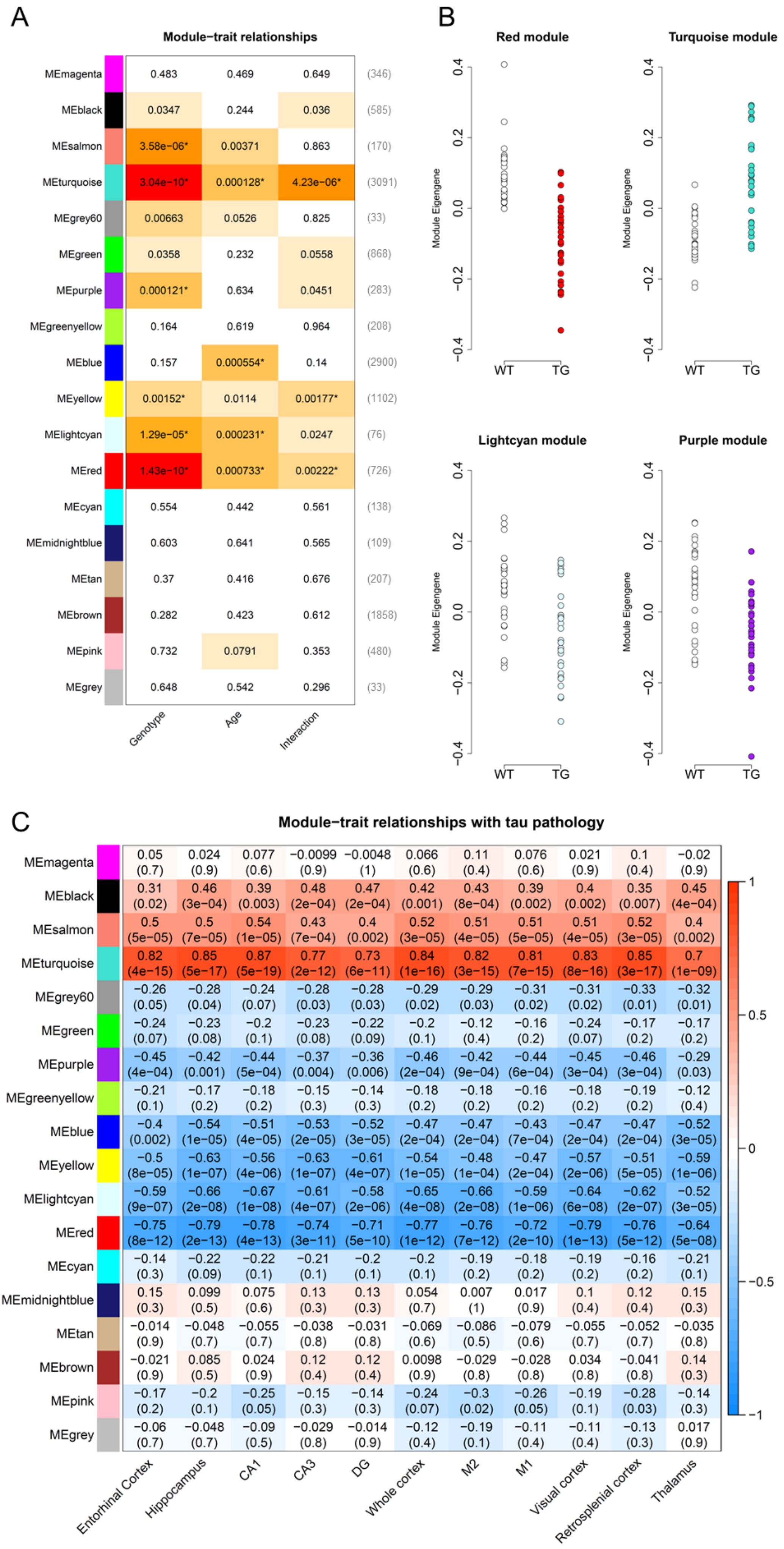
A
Module−trait relationships
MEmagenta 0.483 0.469 0.649 (346)
MEblack 0.0347 0.244 0.036 (585)
MEsalmon 3.58e−06* 0.00371 0.863 (170)
MEturquoise 3.04e−10* 0.000128* 4.23e−06* (3091)
MEgrey60 0.00663 0.0526 0.825 (33)
MEgreen 0.0358 0.232 0.0558 (868)
MEpurple 0.000121* 0.634 0.0451 (283)
MEgreenyellow 0.164 0.619 0.964 (208)
MEblue 0.157 0.000554* 0.14 (2900)
MEyellow 0.00152* 0.0114 0.00177* (1102)
MElightcyan 1.29e−05* 0.000231* 0.0247 (76)
MEred 1.43e−10* 0.000733* 0.00222* (726)
MEcyan 0.554 0.442 0.561 (138)
MEmidnightblue 0.603 0.641 0.565 (109)
MEtan 0.37 0.416 0.676 (207)
MEbrown 0.282 0.423 0.612 (1858)
MEpink 0.732 0.0791 0.353 (480)
MEgrey 0.648 0.542 0.296 (33)
Genotype
Age
Interaction
B
Red module
Turquoise module
Salmon module
Lightcyan module
Purple module
Yellow module
Module Eigengene
WT
TG
C
Module−trait relationships with tau pathology
MEmagenta 0.05 (0.7) 0.024 (0.9) 0.077 (0.6) −0.0099 (0.9) −0.0048 (1) 0.066 (0.6) 0.11 (0.4) 0.076 (0.6) 0.021 (0.9) 0.1 (0.4) −0.02 (0.9)
MEblack 0.31 (0.02) 0.46 (3e−04) 0.39 (0.003) 0.48 (2e−04) 0.47 (2e−04) 0.42 (0.001) 0.43 (8e−04) 0.39 (0.002) 0.4 (0.002) 0.35 (0.007) 0.45 (4e−04)
MEsalmon 0.5 (5e−05) 0.5 (7e−05) 0.54 (1e−05) 0.43 (7e−04) 0.4 (0.002) 0.52 (3e−05) 0.51 (4e−05) 0.51 (5e−05) 0.51 (4e−05) 0.52 (3e−05) 0.4 (0.002)
MEturquoise 0.82 (4e−15) 0.85 (5e−17) 0.87 (5e−19) 0.77 (2e−12) 0.73 (6e−11) 0.84 (1e−16) 0.82 (3e−15) 0.81 (7e−15) 0.83 (8e−16) 0.85 (3e−17) 0.7 (1e−09)
MEgrey60 −0.26 (0.05) −0.28 (0.04) −0.24 (0.07) −0.28 (0.03) −0.28 (0.03) −0.29 (0.02) −0.29 (0.03) −0.31 (0.02) −0.31 (0.02) −0.33 (0.01) −0.32 (0.01)
MEgreen −0.24 (0.07) −0.23 (0.08) −0.2 (0.1) −0.23 (0.08) −0.22 (0.09) −0.2 (0.1) −0.12 (0.4) −0.16 (0.2) −0.24 (0.07) −0.17 (0.2) −0.17 (0.2)
MEpurple −0.45 (4e−04) −0.42 (0.001) −0.44 (5e−04) −0.37 (0.004) −0.36 (0.006) −0.46 (2e−04) −0.42 (9e−04) −0.44 (6e−04) −0.45 (3e−04) −0.46 (3e−04) −0.29 (0.03)
MEgreenyellow −0.21 (0.1) −0.17 (0.2) −0.18 (0.2) −0.15 (0.3) −0.14 (0.3) −0.18 (0.2) −0.18 (0.2) −0.16 (0.2) −0.18 (0.2) −0.19 (0.2) −0.12 (0.4)
MEblue −0.4 (0.002) −0.54 (1e−05) −0.51 (4e−05) −0.53 (2e−05) −0.52 (3e−05) −0.47 (2e−04) −0.47 (2e−04) −0.43 (7e−04) −0.47 (2e−04) −0.47 (2e−04) −0.52 (3e−05)
MEyellow −0.5 (8e−05) −0.63 (1e−07) −0.56 (4e−06) −0.63 (1e−07) −0.61 (4e−07) −0.54 (1e−05) −0.48 (1e−04) −0.47 (2e−04) −0.57 (2e−06) −0.51 (5e−05) −0.59 (1e−06)
MElightcyan −0.59 (9e−07) −0.66 (2e−08) −0.67 (1e−08) −0.61 (4e−07) −0.58 (2e−06) −0.65 (4e−08) −0.66 (2e−08) −0.59 (1e−06) −0.64 (6e−08) −0.62 (2e−07) −0.52 (3e−05)
MEred −0.75 (8e−12) −0.79 (2e−13) −0.78 (4e−13) −0.74 (3e−11) −0.71 (5e−10) −0.77 (1e−12) −0.76 (7e−12) −0.72 (2e−10) −0.79 (1e−13) −0.76 (5e−12) −0.64 (5e−08)
MEcyan −0.14 (0.3) −0.22 (0.09) −0.22 (0.1) −0.21 (0.1) −0.2 (0.1) −0.2 (0.1) −0.19 (0.2) −0.18 (0.2) −0.19 (0.2) −0.16 (0.2) −0.21 (0.1)
MEmidnightblue 0.15 (0.3) 0.099 (0.5) 0.075 (0.6) 0.13 (0.3) 0.13 (0.3) 0.054 (0.7) 0.007 (1) 0.017 (0.9) 0.1 (0.4) 0.12 (0.4) 0.15 (0.3)
MEtan −0.014 (0.9) −0.048 (0.7) −0.055 (0.7) −0.038 (0.8) −0.031 (0.8) −0.069 (0.6) −0.086 (0.5) −0.079 (0.6) −0.055 (0.7) −0.052 (0.7) −0.035 (0.8)
MEbrown −0.021 (0.9) 0.085 (0.5) 0.024 (0.9) 0.12 (0.4) 0.12 (0.4) 0.0098 (0.9) −0.029 (0.8) −0.028 (0.8) 0.034 (0.8) −0.041 (0.8) 0.14 (0.3)
MEpink −0.17 (0.2) −0.2 (0.1) −0.25 (0.05) −0.15 (0.3) −0.14 (0.3) −0.24 (0.07) −0.3 (0.02) −0.26 (0.05) −0.19 (0.1) −0.28 (0.03) −0.14 (0.3)
MEgrey −0.06 (0.7) −0.048 (0.7) −0.09 (0.5) −0.029 (0.8) −0.014 (0.9) −0.12 (0.4) −0.19 (0.1) −0.11 (0.4) −0.11 (0.4) −0.13 (0.3) 0.017 (0.9)
Entorhinal Cortex
Hippocampus
CA1
CA3
DG
Whole cortex
M2
M1
Visual cortex
Retrosplenial cortex
Thalamus
1
0.5
0
−0.5
−1

Figure S9. Co-expression modules associated with tau pathology in rTg4510 mice, related to Figure 6. In total 18 co-expression modules (each labelled with an arbitrary color) were identified in the rTg4510 TG and WT mice (n = 58 RNA-seq datasets). **(A)** Shown is the association of each module with genotype, age and progressive changes in TG mice (i.e. the interaction between genotype and age). Bonferroni corrected significant (α=0.05) *P* values are highlighted with an asterisk. The total number of genes in each module is shown in parentheses. **(B)** Entorhinal cortex co-expression modules associated with rTg4510 genotype. Six modules were significantly different (Bonferroni corrected $P < 0.05$) between TG and WT mice: Red module (linear regression, $t(53) = -7.93$, $\beta = -0.18$, $P = 1.43E-10$), Turquoise module (linear regression, $t(53) = 7.73$, $\beta = 0.18$, $P = 3.04E-10$). **(c)** Salmon module (linear regression, $t(53) = 5.17$, $\beta = 0.14$, $P = 3.58E-06$), Light-cyan module (linear regression, $t(53) = -4.81$, $\beta = -0.13$, $P = 1.29E-05$), Purple module (linear regression, $t(53) = -4.15$, $\beta = -0.13$, $P = 0.00012$), and Yellow module (linear regression, $t(53) = -3.34$, $\beta = -0.10$, $P = 0.0015$). Shown for each module is the module eigengene value for each individual animal (total n = 58 mice). Coloured circles represent rTg4510 TG mice and white circles represent WT control mice. Each circle represents a single mouse. **(C)** Entorhinal cortex co-expression modules are strongly correlated with tau pathology across multiple brain regions. Heatmap representing module-trait relationships between module eigengenes and tau pathology measured using immunohistochemistry in each brain region assessed, where red indicates a positive correlation and blue indicates a negative correlation. Values indicate correlation coefficients and their corresponding *P* values in parentheses. Total n = 58 animals (6-8 animals per group).

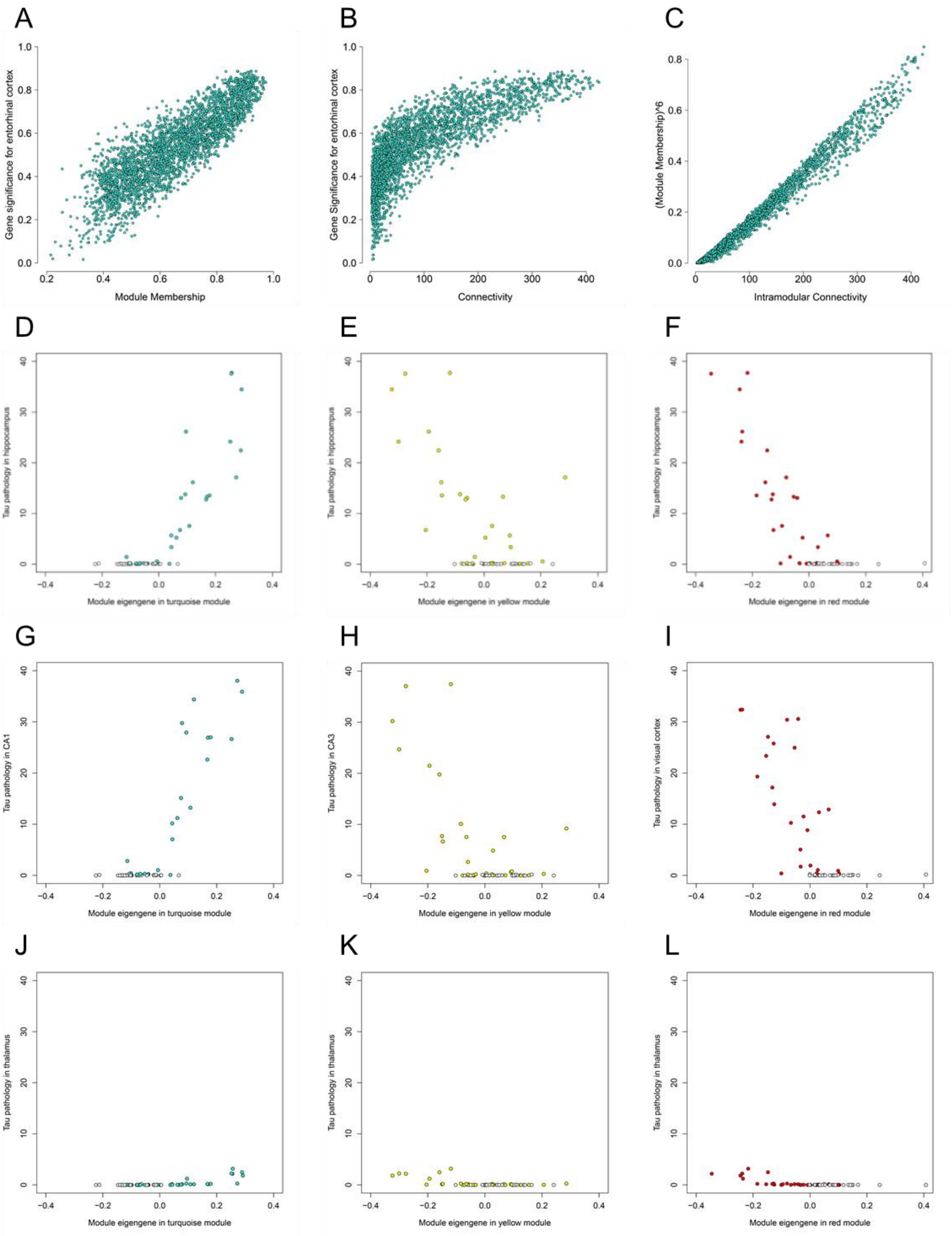

Figure S10. Highly connected transcripts in the three co-expression networks showing progressive changes in rTg4510 mice are most strongly correlated with pathological burden measured by immunohistochemistry in regions of the brain affected early in AD, related to Figure 6. (A) Correlation of module membership for each gene with tau pathology in the entorhinal cortex in the turquoise module (n = 3091 transcripts, Pearson correlation, r = 0.86, P < 1.00E-200). **(B)** Correlation of intramodular connectivity for each gene with tau pathology in the entorhinal cortex in the turquoise module (Pearson correlation, r = 0.81, P < 1.00E-200). **(C)** Module membership raised to power 6 (y-axis) against intramodular connectivity (x-axis) in turquoise module (Pearson correlation, r = 0.99, P < 1.00E-200). **(D)** Correlation between module eigengene in the turquoise module and

tau pathology in the hippocampus (Pearson correlation, r = 0.85). **(E)** Correlation between module eigengene in the yellow module and tau pathology in the hippocampus (Pearson correlation, r = -0.63). **(F)** Correlation between module eigengene in the red module and tau pathology in the hippocampus (Pearson correlation, r = -0.79). **(G)** Correlation of module eigengenes for each sample with tau pathology in the CA1 subregion of the hippocampus in the turquoise module (Pearson correlation, r = 0.87). **(H)** Correlation of module eigengenes for each sample with tau pathology in the CA3 subregion of the hippocampus in the yellow module (Pearson correlation, r = -0.63). **(I)** Correlation of module eigengenes for each sample with tau pathology in the visual cortex in the red module (Pearson correlation, r = -0.79). **(J)** Correlation of module eigengenes for each sample with tau pathology in the thalamus in the turquoise module (Pearson correlation, r = 0.70). **(K)** Correlation of module eigengenes for each sample with tau pathology in the thalamus in the yellow module (Pearson correlation, r = -0.59). **(L)** Correlation of module eigengenes for each sample with tau pathology in the thalamus in the red module (Pearson correlation, r = -0.64). Correlations and corresponding *P* values for all modules and traits can be seen in **Figure S9C**. **(A-C)** Each circle represents a single gene. **(D-L)** Each circle represents a single individual mouse, with coloured circles representing rTg4510 TG mice and white circles representing WT control mice. Total n = 58 animals (6-8 animals per group).
